# Syringeable immunotherapeutic nanogel reshapes tumor microenvironment and prevents tumor metastasis and recurrence

**DOI:** 10.1038/s41467-019-11730-8

**Published:** 2019-08-20

**Authors:** Chanyoung Song, Hathaichanok Phuengkham, Young Seob Kim, Van Vuong Dinh, Inho Lee, Il Woo Shin, Hong Sik Shin, Seung Mo Jin, Soong Ho Um, Hyunseung Lee, Kwan Soo Hong, Seon-Mi Jin, Eunji Lee, Tae Heung Kang, Yeong-Min Park, Yong Taik Lim

**Affiliations:** 1 Department of Nano Engineering, SKKU Advanced Institute of Nanotechnology (SAINT), and School of Chemical Engineering, Biomedical Institute for Convergence at SKKU, Sungkyunkwan University, 2066 Seobu-ro, Jangan-gu, Suwon, Gyeonggi-do 16419 Republic of Korea; 2 Department of Immunology, School of Medicine, Konkuk University, 268, Chungwondaero, Chungju-si, Chungcheongbuk-do Republic of Korea; 3 Bioimaging Research Team, Korea Basic Science Institute, Cheongju, 28119 Republic of Korea; 4 School of Materials Science and Engineering, Gwangju Institute of Science and Technology, Gwangju, 61005 Republic of Korea

**Keywords:** Cancer immunotherapy, Drug development

## Abstract

The low response rate of current cancer immunotherapy suggests the presence of few antigen-specific T cells and a high number of immunosuppressive factors in tumor microenvironment (TME). Here, we develop a syringeable immunomodulatory multidomain nanogel (iGel) that overcomes the limitation by reprogramming of the pro-tumoral TME to antitumoral immune niches. Local and extended release of immunomodulatory drugs from iGel deplete immunosuppressive cells, while inducing immunogenic cell death and increased immunogenicity. When iGel is applied as a local postsurgical treatment, both systemic antitumor immunity and a memory T cell response are generated, and the recurrence and metastasis of tumors to lungs and other organs are significantly inhibited. Reshaping of the TME using iGel also reverts non-responding groups to checkpoint blockade therapies into responding groups. The iGel is expected as an immunotherapeutic platform that can reshape immunosuppressive TMEs and synergize cancer immunotherapy with checkpoint therapies, with minimized systemic toxicity.

## Abstract (web summary)

The limited efficacy of current immunotherapy suggests low antigen-specific T cells and immunosuppressive factors in tumor microenvironment (TME). Here, the authors develop a syringeable immunomodulatory multi-domain nanogel that can reprogram the TME and induce enhanced cancer immunotherapy.

## Introduction

Surgery maintains its strong position as a therapeutic modality for treating established solid tumors, and various immunotherapies, such as cancer vaccines, checkpoint therapies, and adoptive T-cell transfer, are applied to treat residual tumors after surgery and prevent tumor recurrence and metastasis^1,2^. However, immunotherapies have a low-response rate, which ranges from ~5–30% depending on the target tumor^3–5^. The low response is related to the presence of few T cells (cold tumors) and a high number of immunosuppressive cells, such as myeloid-derived suppressor cells (MDSCs), tumor-associated macrophages (TAMs) and regulatory T cells (Tregs) in the tumor microenvironment (TME)^6–9^. Although combination cancer immunotherapies that can prime antitumor immune responses as well as modulation of immunosuppression have been adopted in the clinic to induce synergistic therapeutic efficacy, conventional systemic delivery-based combinations could abrogate the therapeutic effects of immunotherapies, and induce severe systemic and off-target immune toxicities^10–14^. Therefore, a new therapeutic strategy that can overcome the limitations of current immunotherapies by modulating tumor-induced immunosuppression in the TME and improve therapeutic efficacy with minimized toxicity is highly required^8,15–20^. The key features of nanoparticle size, shape, and surface molecule organization have been used to induce innate immune responses that promote adaptive immunity^21–28^. Different nanoparticle strategies have also been investigated to target MDSCs and TAMs for the treatment of cancer, such as the inhibition of their recruitment and activation, their depletion by inducing apoptosis or by promoting their differentiation into mature cells that are non-suppressive^29–31^. However, the limitation of nanoparticles in systemic and off-target immune toxicities has frustrated the clinical advancement of many nanoparticle formulations for the systemic delivery of immunomodulatory drugs. In contrast, local delivery can increase the bioavailability and allow multiple combination therapies of immunostimulatory drugs, while preventing significant systemic exposure and off-target toxicities^32–34^. To address this issue, we hypothesized that the local synthetic immune niches based on nanopharmaceutical formulations of multiple immunomodulatory drugs for the modulating key components of the TME could be used to overcome the abovementioned limitations of current immunotherapy.

**Fig. 1:**
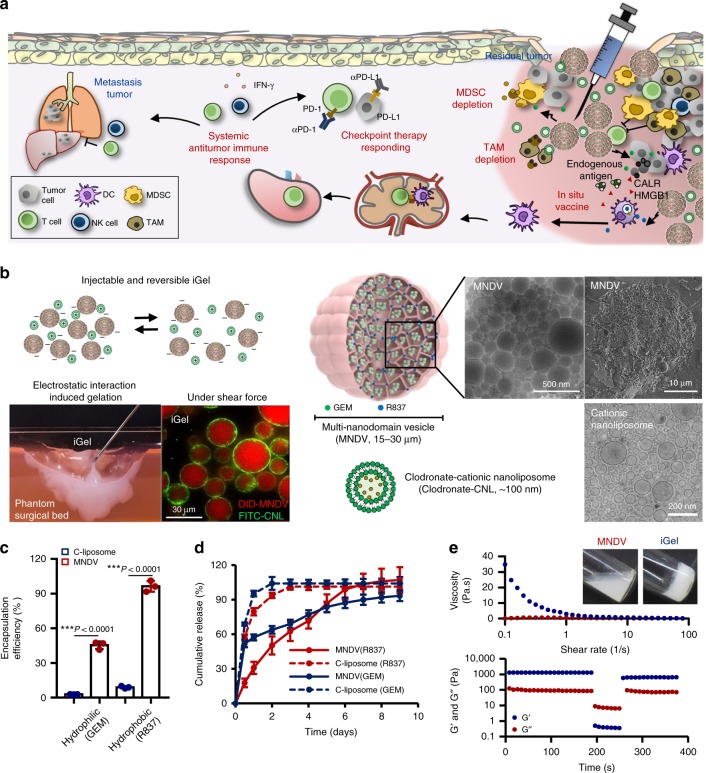
MNDV and syringeable iGel for post surgical local treatment. **a** Schematic depiction of iGel for the prevention of post operative tumor recurrence and metastasis. Gemcitabine (GEM) and clodronate-CNLs act as MDSC-, and TAM-depleting drugs to revert the immunosuppressive microenvironment. Release of the vaccine, R837, and an endogenous antigen provide immunostimulation and elicit an antitumor immune response. Moreover, combination treatment with checkpoint therapy can help to boost the immune response against cancer cells. **b** Left: schematic illustration of injectable and reversible iGel. A photograph image represents the characteristic of iGel in easy injectability onto irregular cavity in phantom surgical bed and fluorescent images of the gel showing that DID-loaded MNDVs were interconnected by FITC-labeled CNLs. Right: schematic of MNDV and CNL. The inset shows a transmission and scanning electron cryomicrographs of the structure of MNDVs and CNLs. **c** Comparison of hydrophilic (GEM) and hydrophobic (R837) drug encapsulation efficiency in MNDVs versus conventional liposomes (C-liposomes). **d** Kinetics of drug release from MNDVs and C-liposomes. *P-*values were analyzed by Student’s *t*-test (*n* = 3). **e** Rheological properties of iGel. Viscosity over shear rates of MNDVs versus the gel (upper). Recovery of the gel undergoing cyclic deformation of 0.2 and 500% strain with *G*′ and *G*″ (lower). The results are representative of one of three independent experiments. Source data are provided as a Source Data file

Here, we suggest a syringeable synthetic immune niche based on nanoliposome-bridged non-concentric multi-nanodomain vesicle gels that can modulate tumor-induced immunosuppressive TMEs in a spatiotemporal manner, enhance antitumor immune priming as an in situ cancer vaccine, and increase the therapeutic efficacy of combination cancer therapies with checkpoint therapies while minimizing treatment-associated adverse effects (Fig. 1a). The immunomodulatory multidomain nanogel (iGel) can be formed by electrostatic interactions with negatively charged non-concentric multi-nanodomain vesicles (MNDVs) and positively charged nanoliposomes, which were loaded with immunomodulatory drugs, and the gel structure can be temporally disassembled by shear-force during the syringe injection and assembled again after the shear-force is removed at the treatment site (Fig. 1b). Non-concentric MNDVs significantly increase the encapsulation efficiency and release time of both hydrophilic gemcitabine and hydrophobic imiquimod (R837), and reshape the TME via the immunogenic cell death (ICD) of tumor cells, activation of recruited antigen-presenting cells (APCs), generation of antigen-specific T cells, and depletion of inhibitory MDSCs^35–38^. The clodronate-cationic nanoliposomes (clodronate-CNLs) that interconnect MNDVs and induce shear-force-dependent reversible gel also deplete inhibitory TAMs^39^. Local reprogramming of the TME by iGel leads to systemic antitumor immunity and synergizes the therapeutic efficacy of immune checkpoint inhibitors, resulting in the prevention of tumor recurrence and metastasis (Fig. 1a).

## Results

### Characterization and fabrication of MNDV and iGel

Figure 1b shows a transmission electron cryomicrographs of the internal structure of MNDVs and CNLs that were used to form injectable and reversible iGel. Compared with conventional multivesicular liposome where micro-sized aqueous compartments were interconnected, nano-sized multidomain vesicle was observed in MNDV (Supplementary Fig. 1). Furthermore, MNDVs are characterized by their unique structure consisting of multiple non-concentric aqueous compartments surrounded by a network of lipid membranes (Fig. 1b and Supplementary Fig. 2), which are different from that of traditional liposomes. Confocal image also illustrates the structure and morphology at the microscopic level of a fluorescent double-labeled MNDV composed of hydrophilic (loaded with fluorescein isothiocyanate (FITC) protein) and hydrophobic (loaded with DID) compartments. The distribution of hydrophobic and hydrophilic dyes in MNDVs shows no preferential segregation of either molecule in MNDVs (Supplementary Fig. 3). The unique structure rendered by the introduction of oils (triolein, squalene and oleic acid) as biological glue between the hydrophobic lipid layers of each nanodomain provided a high amount and simultaneous loading of both hydrophilic and hydrophobic drugs as well as enhanced structural stability (Fig. 1c and Supplementary Fig. 4). The long-term storage stability of MNDVs was evaluated at 4 °C and 37 °C; they could maintain their size of ~30 µm for over a month (Supplementary Fig. 5). Although various types of nanoliposome formulations have been utilized as drug delivery vehicles for hydrophilic substances, they have low loading efficacy and stability^40^. However, the distinct structure of MNDVs could enable the high loading of hydrophilic substances, as well as the sustained release of encapsulated drugs. The loading efficiency of both hydrophilic gemcitabine and hydrophobic R837 was increased by 16.1- and 10.8-fold, respectively, for MNDVs compared with that for conventional liposomes (C-liposomes) with the same lipid composition (Fig. 1c). The release of encapsulated gemcitabine and R837 from MNDVs was also extended over a prolonged period (Fig. 1d) compared with that from C-liposomes. MNDVs were further developed as a syringeable gel that can fill the irregular space of a tumor surgical bed without dead volume (Fig. 1b), prolong the residence time of MNDVs, and extend the release of immunomodulatory drugs in the surgical area without moving into other sites. To fabricate the MNDV-based syringeable gel, we adopted CNLs that can be easily assembled with anionic MNDVs via a mutual electrostatic attraction. The optimal mixing ratio (10:3 by weight) between MNDVs and CNLs was selected based on gel stability (Supplementary Fig. 6). The non-flowing characteristic (Fig. 1e) and fluorescence images (Fig. 1b and Supplementary Fig. 7) of MNDVs mixed with CNLs suggested electrostatic interaction-driven gel (iGel) formation. iGel also exhibited a much higher viscosity (~35 Pa s) than did MNDVs alone (~0.1 Pa s) and showed excellent shear thinning, which would allow the gel to be injected through a fine syringe but revert to a gel upon removal of the stress and be localized to the treatment site (Fig. 1e). The storage and loss moduli of iGel decreased when shear-force was applied, which recovered immediately after the shear-force was removed (Fig. 1e). The scanning electron microscopy image of lyophilized iGel also suggested that MNDV vesicles were interconnected (Supplementary Fig. 7). The long-term stability of iGel was evaluated, which showed stable for 4 and 3 weeks after incubation in 4 and 37 °C, respectively (Supplementary Fig. 8)^41,42^.

### Extended-release iGel formulation reduces systemic toxicity

The release of gemcitabine and R837 from iGel was prolonged over a week, as shown in Supplementary Fig. 9a, b, suggesting that the extended drug release properties of iGel were driven by the unique structure of MNDVs. As the separation of CNLs from iGel plays a crucial role in maintaining the stability and retention of iGel, their release behavior was assessed after they were labeled with FITC. CNLs were gradually released from iGel over 2 weeks, as shown in Supplementary Fig. 9c.

**Fig. 2:**
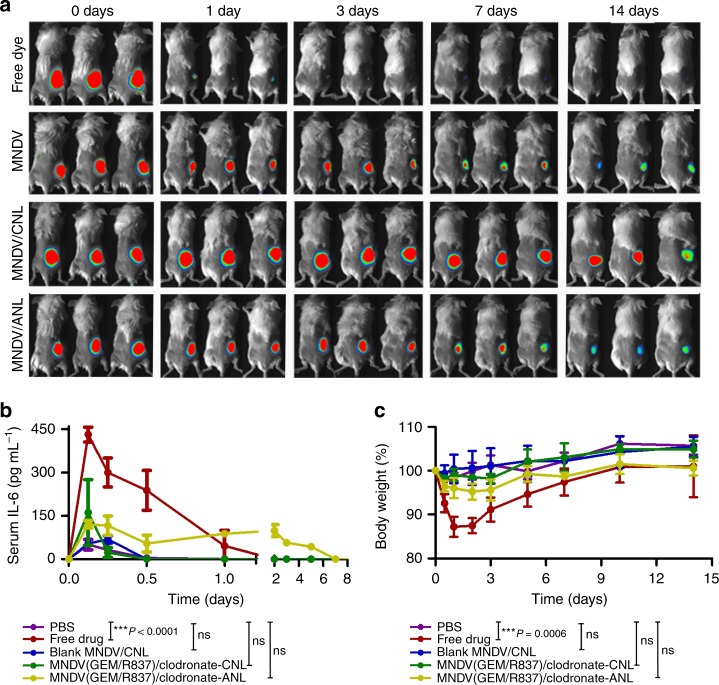
Extended release of drugs from localized iGel can reduce systemic toxicity. **a** Fluorescence imaging depicting the retention of IR dye-labeled MNDVs after treatment. **b** Serum levels of IL-6 and **c** percentage of body weight change following subcutaneous administration of different treatment groups. Data are presented as the means ± S.D. (*n* = 4). *P*-values were analyzed by one-way ANOVA and Tukey’s test (serum IL-6 data at 3 h and body weight data at day 1). The results are representative of one of two independent experiments. Source data are provided as a Source Data file

For the in vivo retention of iGel, MNDVs were labeled with IR dye 800 and fabricated iGel was subcutaneously injected into the right flank of mice. The iGel was retained at the injection site over 2 weeks (Fig. 2a and Supplementary Fig. 10), while injected free dye, MNDVs alone and MNDVs/anionic nanoliposomes (ANLs) diffused very rapidly within a few days. We also investigated whether the gel formulation of MNDVs (GEM/R837)/clodronate-CNLs could minimize systemic cytokine (interleukin (IL)-6) and toxicity levels (Fig. 2b)^26,43^. Notably, the local administration of free drugs led to burst leakage into the systemic circulation and induced a high level of IL-6 expression, which resulted in body weight loss in mice (Fig. 2c). In fact, R837 and related analogs have been used for topical treatment of cutaneous cancers in the clinic^44^. However, repetitive systemic administration of R837 induces dose-limiting adverse events (fever and headache), which limit clinical efficacy^45^. The localized and sustained release of R837 with iGel in the injection site was attributed to decreased systemic side effects. Recently, Toll-like receptor 7/8 agonists were conjugated into polymer backbone to decrease systemic toxicity of them^43,46^. However, both the chemical modification of Toll-like receptor agonist such as R837 and subsequent conjugation of it onto synthetic polymer backbone would limit clinical translation because systematic studies for human safety of synthetic materials are necessary. In contrast, all the lipid components and drugs (gemcitabine, R837, and clodronate) used for the syringeable iGel were selected based on their approval or prior use in a clinical setting, indicating the effectiveness of iGel in humans. The concentrations of liver enzymes (alanine aminotransferase (ALT) and aspartate aminotransferase (AST)) and a surrogate marker of kidney function (blood urea nitrogen (BUN)) were confirmed and showed a lack of detectable changes across the treatment groups (Supplementary Fig. 11a)^26,47^. In addition, histopathological examination of the lungs, liver and kidneys confirmed no special toxicity-associated changes in any of the treatment groups (Supplementary Fig. 11b).

### Immunomodulatory function of drugs loaded MNDVs and CNLs

**Fig. 3:**
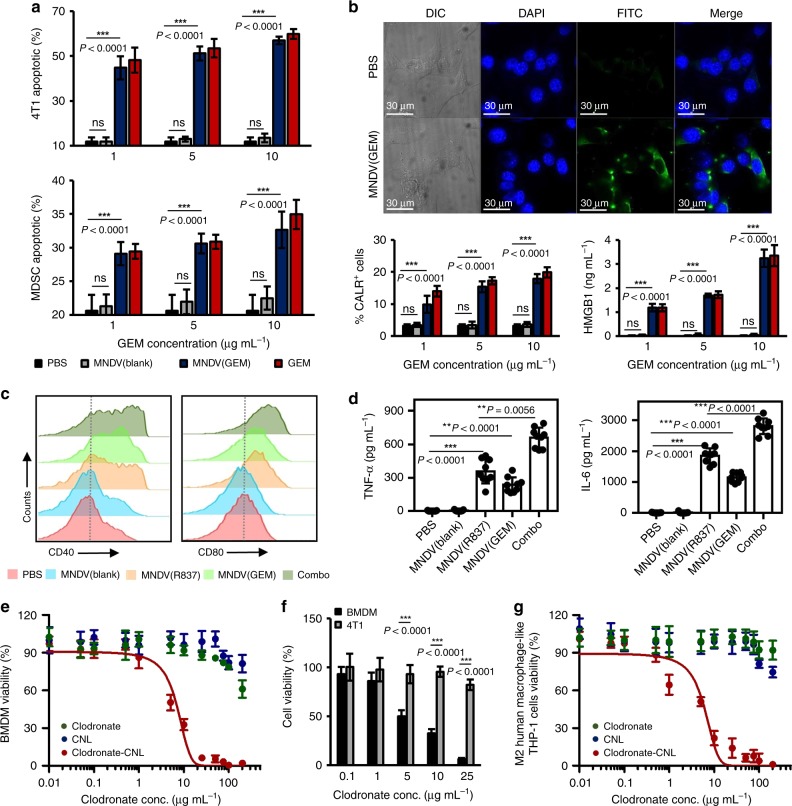
In vitro test of induction of an in situ cancer vaccine and depletion of immunosuppressive cells. **a** In vitro test of MNDV(GEM)-induced apoptosis. Percentage of apoptotic 4T1 cells (upper) and MDSCs (lower) (Annexin-V and PI double-positive cells). **b** MNDV(GEM) induced immunogenic changes in 4T1 cells. Representative fluorescence images showing the induction of CALR expression in 4T1 cells in the presence of MNDV(GEM) for 4 h. The cell nuclei and CALR were detected by Hoechst and anti-CALR/FITC-conjugated anti-IgG antibodies staining, respectively. Flow cytometry analysis of CALR^+^ 4T1 cells. HMGB1 release into the supernatants of MNDV(GEM)-treated 4T1 cell cultures was examined by ELISA 24 h after treatment. **c** Histograms representing the cell surface expression of activation and maturation markers in BMDCs following treatment with MNDV(R837), MNDV(GEM) (MNDV(GEM)-treated 4T1 cell-conditioned media), or Combo. **d** Quantification of IL-6 and TNF-α production via ELISA following the treatment. *P-*values were determined by one-way ANOVA and Tukey’s test. **e** Concentration-dependent cytotoxicity of clodronate-CNLs to BMDMs. **f** Cytotoxicity of clodronate-CNLs to BMDMs and 4T1 cells. *P-*values were determined by Student’s *t*-test. **g** Concentration-dependent cytotoxicity of clodronate-CNLs to human M2 macrophage-like THP-1 cells. Viability of the treated cells was assessed by MTS assay. Data are each pooled from three independent experiments and presented as the mean ± SD (*n* = 9). Source data are provided as a Source Data file

In addition to the conventional usage of gemcitabine as a chemotherapeutic agent, it has the ability to deplete MDSCs^37,38^ and induce ICD^36,48^. The apoptosis of tumor cells and MDSCs by MNDVs loaded with gemcitabine (MNDV (GEM)) was confirmed (Fig. 3a). In contrast, MNDV (GEM) had no significant effect to T cells and bone marrow-derived dendritic cells (BMDCs) around 1 µg mL^−1^ concentration, which expected to be exposed in vivo (Supplementary Fig. 12)^49^. As shown in Fig. 3b and Supplementary Fig. 13a, 4T1 and TC1 cancer cells undergoing ICD upregulated calreticulin (CALR, eat-me signal) expression on the cell surface and secreted high-mobility group box 1 (HMGB1, danger signal) after treatment with MNDV (GEM)^35,36,50^. To test whether the induction of ICD can induce stronger immune responses, we incubated BMDCs with the conditioned media of 4T1 and TC1 cells treated with MNDV (GEM) and MNDV (R837). BMDCs showed unregulated CD40 and CD80 surface marker expression and increased IL-6 and tumor necrosis factor (TNF)-α production (Fig. 3c, d and Supplementary Fig. 13b, c). In addition, cotreatment of MNDV (GEM)-treated tumor cells with MNDV (R837) adjuvant significantly enhanced the activation of BMDCs, which suggested the function of MNDVs loaded with gemcitabine and R837 as an in situ cancer vaccine. To investigate the immune response of MNDV(GEM/R837) in vivo, the therapeutic-loaded MNDVs and soluble drugs were peritumorally injected into the surgical site. Compared with treatment with MNDVs containing gemcitabine or R837 alone or a bolus injection, treatment with MNDVs containing both gemcitabine and R837 also synergistically inhibited tumor growth and lung metastasis (Supplementary Fig. 14). CNLs were prepared and loaded with clodronate as a TAM-depleting agent, as shown in Supplementary Table 1^39,51^. Treatment with clodronate-CNLs was more effective than treatment with free clodronate in killing bone marrow-derived macrophages (BMDMs) in a concentration-dependent manner, resulting in an IC_50_ of 6.7 µg mL^−1^ (Fig. 3e). Furthermore, clodronate-CNLs specifically acted on macrophages (Fig. 3f) rather than on tumor cells. When human cervical cancer and macrophage cell lines were tested to evaluate a potential to be translated into clinic, experimental results showed consistency with those of murine cells. Clodronate-CNLs effectively killed human M2 macrophage-like THP-1 cells in a concentration-dependent manner with IC_50_ of 5.6 µg mL^−1^ (Fig. 3g) and MNDVs loaded with gemcitabine induced apoptosis in human C33a cervical cancer cells (Supplementary Fig. 13d). Cotreatment of gemcitabine-treated tumor cells with R837 adjuvant also significantly increased pro-inflammatory cytokine production in human macrophage-like THP-1 cells (Supplementary Fig. 13e).

### Post surgical treatment with iGel reshapes TME

**Fig. 4:**
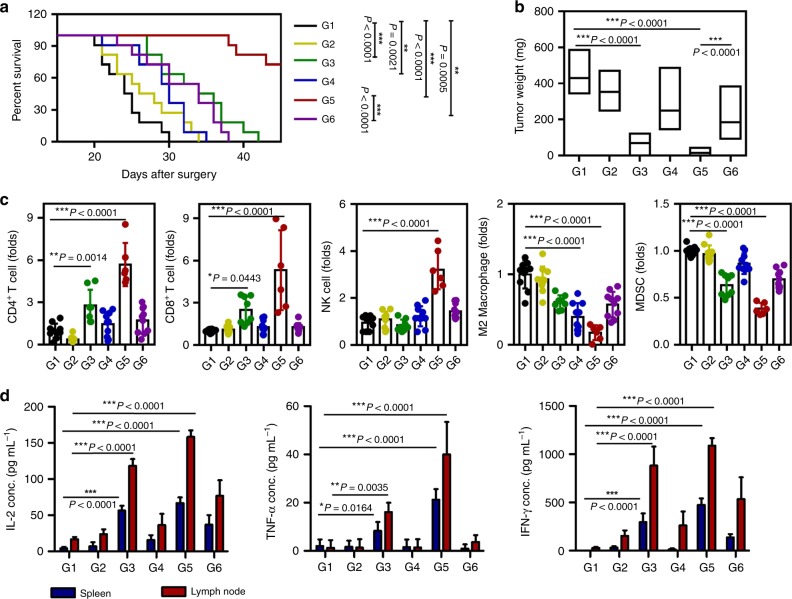
Antitumor effects of syringeable iGel in post surgical 4T1 tumor models. Tumors were resected when the tumor reached 300 mm^3^ in size and were subsequently treated as follows: G1, surgery only; G2, blank gel; G3, MNDV(GEM/R837)/CNL; G4, blank MNDV/clodronate-CNL; G5, MNDV(GEM/R837)/clodronate-CNL; and G6, MNDV(GEM/R837)/clodronate-ANL. **a** Survival rate of mice after treatment. Differences in survival were determined for each group (*n* = 11) by the Kaplan–Meier method, and the overall *P-*value was calculated by the log-rank test. **b** Recurrent tumor weight (*n* = 10). **c** FACSs analysis demonstrating infiltrating CD4^+^, CD8^+^ T cells, NK cells, M2 macrophages, and MDSCs in recurring tumors at day 7 post surgery (*n* = 10). **d** Production of cytokines related to the antigen-specific response in spleen and tumor-draining lymph nodes (*n* = 6). Lymphocytes isolated from the spleen and tumor-draining lymph nodes were stimulated with 4T1 tumor antigens for 72 h. After incubation, the supernatants were collected. IL-2, TNF-α, and IFN-γ production was measured by ELISA. Data are presented as the mean ± SD. Statistical significance was calculated by one-way ANOVA and Tukey’s test. Data are each pooled from two independent experiments for **b**–**d**. Source data are provided as a Source Data file

**Fig. 5:**
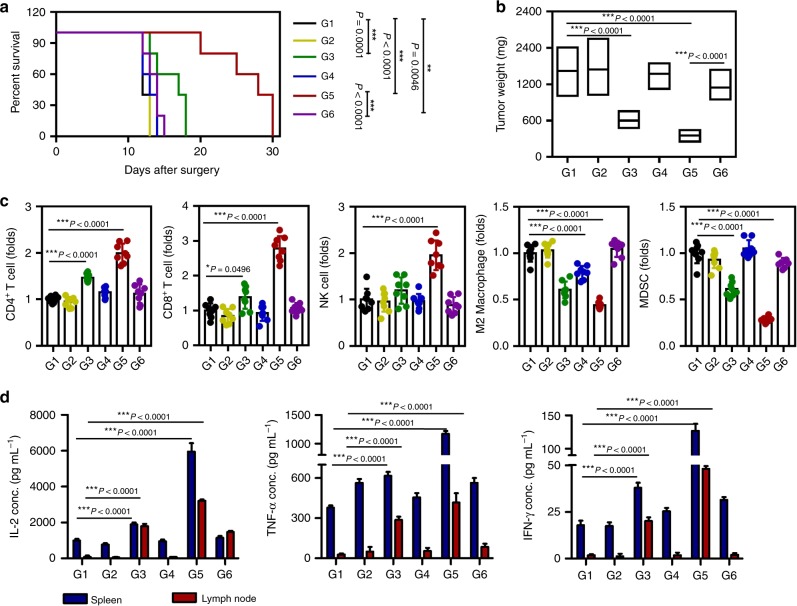
Antitumor effects of syringeable iGel in post surgical TC1 tumor models. Tumors were resected when the tumor reached 300 mm^3^ in size and subsequently treated as indicated in 4T1 tumor model. **a** Survival rate of mice after treatment. Differences in survival were determined for each group (*n* = 10) by the Kaplan–Meier method, and the overall *P*-value was calculated by the log-rank test. **b** Recurrent tumor weight (*n* = 10). **c** FACSs analysis demonstrating infiltrating CD4^+^, CD8^+^ T cells, NK cells, M2 macrophages, and MDSCs in recurring tumors at day 7 post surgery (*n* = 8). **d** Production of cytokines related to the antigen-specific response in spleen and tumor-draining lymph nodes (*n* = 6). Lymphocytes isolated from the spleen and tumor-draining lymph nodes were stimulated with TC1 tumor antigens for 72 h. After incubation, the supernatants were collected. IL-2, TNF-α, and IFN-γ production was measured by ELISA. Data are presented as the mean ± SD. Statistical significance was calculated by one-way ANOVA and Tukey’s test. Data are each pooled from two independent experiments for **b**–**d**. Source data are provided as a Source Data file

Generally, surgical resection alone is rarely curative for advanced-stage cancer due to either local recurrence or metastasis. We assumed a condition where some of the tumor could not be removed completely by the surgeon because the tumor was located in an inaccessible or vulnerable location or had metastasized to various organs. We investigated whether iGel could be applied in the surgical field to modulate immunosuppressive TMEs in favor of T-cell-based antitumor immunity. Assuming that T-cell-based antitumor immunity using iGel described here may be effective against a wide spectrum of cancer types, we used murine 4T1 triple-negative breast cancer and TC1 cervical cancer models for animal experiments. We performed the surgery in the manner shown in Supplementary Fig. 15a, removing approximately 90% of the primary tumor (i.e., 10% of the remaining tumor) and injecting iGel into the dissected empty space (i.e., peritumoral injection). In this procedure, mice with the same sized tumors were used for surgery, and mice with the same size residual tumor were carefully selected for further experiments to prevent errors caused by surgery (Supplementary Fig. 15b). All mice treated solely with surgery developed substantial tumors and died within 30 and 13 days after the resection of 4T1 and TC1 tumors, respectively (Figs. 4a, b and 5a, b). The survival rate of mice treated with iGel (G5) was significantly prolonged and notably longer than that of mice injected with the same therapeutic components in the non-gelling formulation (G6). After treatment with iGel, the original TMEs reverted into the TMEs of immunogenic tumors characterized by increased levels of infiltrating CD4^+^, CD8^+^ T cells and natural killer (NK) cells (Figs. 4c and 5c and Supplementary Fig. 16) and upregulated expression of type 1T helper cytokines (TNF-α, IL-6, IFN-γ, and IFN-α) (Supplementary Fig. 17). Moreover, the percentages of MDSCs and M2 macrophages that were involved in the suppressive role in the TME were significantly decreased after treatment with iGel (Figs. 4c and 5c). Interestingly, the local release of therapeutics led to an increase in the percentages of effector cells and a decrease in the percentages of suppressive immune cells in the spleen at day 7 post surgery (Supplementary Fig. 18a). The infiltration of innate immune cells in lymphoid tissue was also observed (Supplementary Fig. 18b), while the percentage of Tregs, which exhibit high infiltration under post surgical conditions and are associated with a poor clinical outcome^6^, was significantly depleted after treatment with iGel (Supplementary Fig. 18c). To identify tumor-specific immune responses, we isolated lymphocytes from the spleen and lymph nodes and then stimulated them with tumor lysates. The highest levels of cytokines associated with the T-cell response, such as IFN-γ, TNF-α, and IL-2, were measured in the G5 group (Figs. 4d and 5d). These experimental results suggested that the iGel containing gemcitabine, R837 and clodronate acted as an immune priming niche to recruit and educate APCs as well as an antitumoral immune niche to enable efficient T-cell activities while minimizing the inhibitory activities of MDSCs and M2 macrophages.

### Local treatment with iGel induces systemic antitumor immunity

**Fig. 6:**
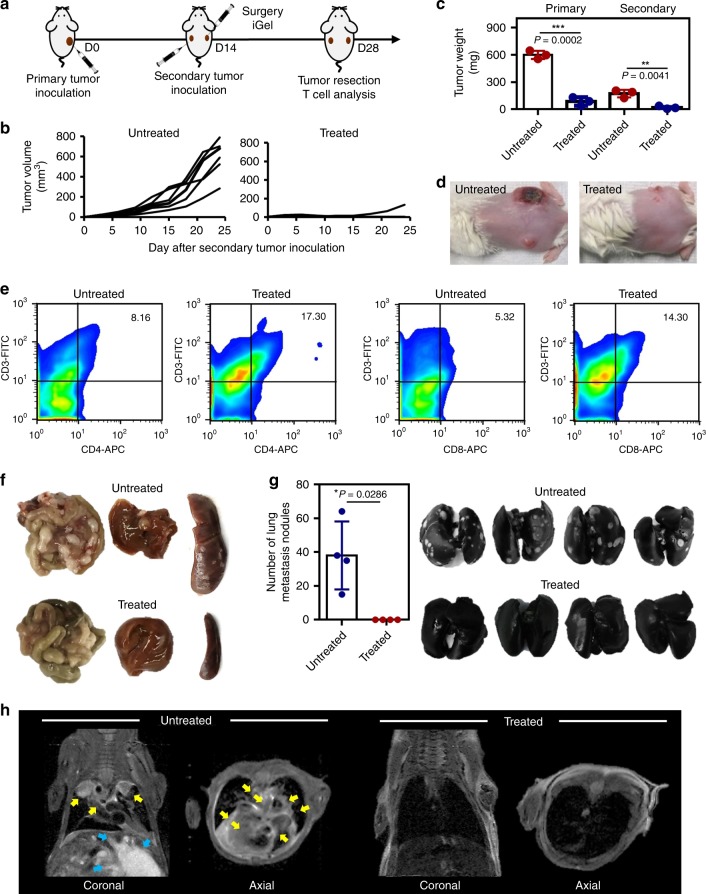
Generation of a systemic antitumor immune response by iGel treatment. **a** Schedule for the systemic antitumor immune response test (*n* = 9, 6 mice were used for **b** and 3 mice were used for **c**–**e**). **b** Secondary tumor growth curves. **c** Tumor weight and **d** representative photographs of mice on day 14 after treatment. Statistical analysis was performed by Student’s *t*-test. **e** Percentages and representative dot plots of CD4^+^ and CD8^+^ T cells in secondary tumors of untreated and treated mice. **f** Representative images of the intestine, liver, and spleen collected from mice 21 days after surgery. **g** Images of lungs collected from mice 21 days after surgery. Number of metastatic lung nodules (*n* = 4). Statistical analysis was performed by Mann–Whitney test. **h** MRI scans images showing tumor metastasis. Yellow arrows in coronal and axial images indicate the tumors in lung. Blue arrows in coronal image of untreated group indicate tumors in other body parts. Another hyper-intense white signal that is not indicated by arrows is related to massive ascites, which reflected the aggressive features of peritoneal spread tumor (*n* = 3). The results are representative of one of two independent experiments. Source data are provided as a Source Data file

To confirm whether the systemic antitumor immune response could be induced by locally injecting iGel, we inoculated mice with cells for secondary tumor formation contralateral to the primary tumor on the same day as the treatment (Fig. 6a). We expected that the growth of the secondary tumor would be suppressed by the increased immune cell infiltration in iGel-treated sites, which could subsequently generate a systemic antitumor immune response. The growth of the secondary tumor was inhibited (Fig. 6b–d), and the population of infiltrating CD4^+^ and CD8^+^ T cells in the secondary tumor was increased by 2.1- and 2.7-times, respectively (Fig. 6e), in the treated groups, suggesting that the systemic antitumor immune response induced by local treatment with iGel inhibited metastasis. Spontaneous metastatic tumor nodules in other organs (intestine, liver and spleen) were detected in the non-treated group, while no noticeable signs of metastasis were detected in the iGel-treated group (Fig. 6f and Supplementary Fig. 19a). We identified significant differences in lung metastasis between the non-treated and treated groups, as shown in Fig. 6g and Supplementary Fig. 19b. When the number of metastatic nodules in the lungs was counted after staining with India ink, we found that iGel could completely inhibit the spontaneous metastasis of tumor cells from the primary tumor to the lungs. Furthermore, tumor metastasis to lung was also detected in a clinically relevant magnetic resonance imaging (MRI) trafficking study (Fig. 6h and Supplementary Fig. 20). Taken together, these results suggested that the systemic antitumor immune response induced by local treatment with iGel inhibited metastasis.

### Local treatment with iGel generates a memory T-cell response

**Fig. 7:**
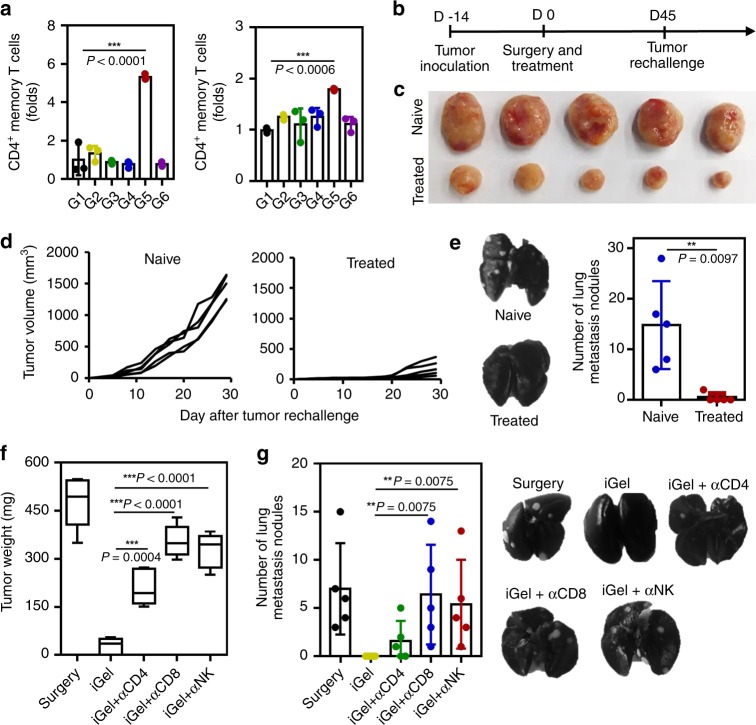
Syringeable iGel for memory T-cell response. **a** Splenocytes isolated from tumor-bearing mice were analyzed for the presence of memory T cells gated on CD4^+^ and CD8^+^ cells (*n* = 3). **b** Treatment schedule for the tumor rechallenge experiment. **c** Tumor image and **d** tumor volume of naive and treated (tumor-free mice after iGel treatment, G5 group) mice. **e** Representative images of lungs collected from mice 30 days after tumor rechallenge. White nodules indicate metastatic tumors in the lungs. Number of metastatic lung nodules (*n* = 5). **f** Weight of recurring tumors after treatment with iGel. Specific immune cell subsets were depleted using anti-CD4 (αCD4), anti-CD8 (αCD8) and anti-CD49b (αNK) antibodies to reveal their relative contributions (*n* = 5). **g** Number of metastatic lung nodules. Data are presented as the mean ± SD (*n* = 5). For **a** and **f**, *P-*values were determined by one-way ANOVA and Tukey’s test. For **e** and **g**, *P*-values were determined by Mann–Whitney test. The results are representative of one of two independent experiments. Source data are provided as a Source Data file

To investigate the effect of local iGel treatment on the memory T-cell response in vivo, we used a tumor rechallenge model (tumor-free mice surviving from a previous survival rate test (Fig. 4) and naive mice). CD4^+^ and CD8^+^ memory T cells (CD44^+^CD62L^+^ gated on CD4^+^ and CD8^+^ cells) were significantly increased in the G5 group only, and we hypothesized that an inhibitory effect on tumor growth could be induced by memory T cells (Fig. 7a)^52^. When we again inoculated mice with 4T1 cells (5 × 10^5^) contralateral to the primary tumor site 45 days after tumor resection and treatment (Fig. 7b), tumor growth in re-challenged G5 mice was inhibited up to 20 days, while continuous tumor growth was observed in naive mice (Fig. 7c, d). Tumor metastasis to the lungs was also significantly inhibited in iGel-treated mice even at 30 days after the tumor rechallenge, strongly suggesting that the antitumor effects of memory T cells were induced by local treatment with iGel (Fig. 7e). To investigate the effects of innate and adaptive immunity on the final antitumor immune responses, we depleted CD4^+^, CD8^+^ and NK cells with a monoclonal antibody at 14 days after tumor cell inoculation and surgery in the 4T1 tumor model. The inhibition of tumor growth and metastasis in mice treated with anti-CD4 (αCD4), anti-CD8 (αCD8) and anti-CD49b (αNK) antibodies, was reduced compared with that in non-depleted mice (Fig. 7f, g). The experimental results suggested that iGel could induce a strong innate and adaptive antitumor immune response and that both NK and T cells are important.

### iGel synergizes therapeutic response of checkpoint therapy

**Fig. 8:**
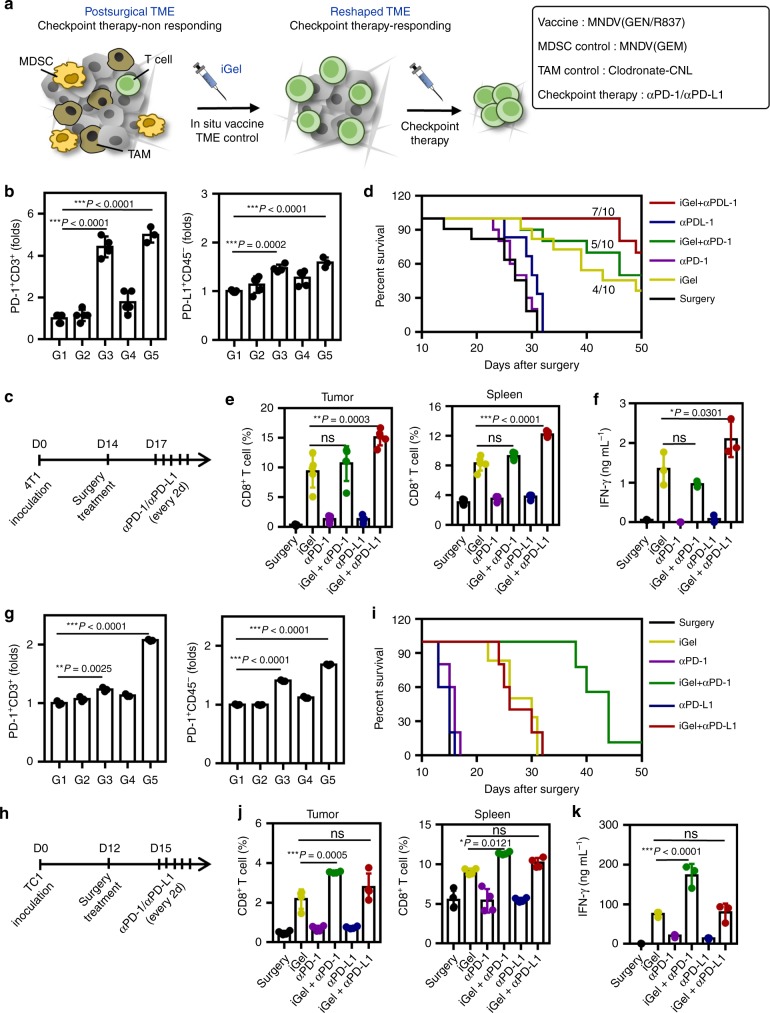
Therapeutic effects of iGel in enhancing antitumor immune response to checkpoint inhibitors in vivo. **a** Schematic depiction of utilizing a syringeable synthetic immune niche based on iGel that can modulate tumor-induced immunosuppressive TMEs in a spatiotemporal manner, enhance antitumor immune priming and turn checkpoint therapy-non-responding tumors into checkpoint therapy-responding tumors. The therapeutic response was tested in the 4T1 (**b**–**f**) and TC1 (**g**–**k**) models. **b** and **g** Upregulation of PD-1 and PD-L1 expression in recurring tumors after treatment (*n* = 5 for 4T1 model *n* = 3 for TC1 model). **c** and **h** Treatment schedule for combination with a checkpoint inhibitor. **d** and **i** Survival curves for treated and control mice (*n* = 10). **e** and **j** Infiltrating CD8^+^ T cells (*n* = 5 for 4T1 model *n* = 4 for TC1 model), and **f** and **k** IFN-γ secretion after combination with checkpoint inhibitors (*n* = 3). Data are presented as the mean ± SD. *P-*values were determined by one-way ANOVA and Tukey’s test. The results are representative of one of two independent experiments. Source data are provided as a Source Data file

The effect of iGel in reshaping the TME on the therapeutic response to checkpoint therapy was investigated using two different antibodies (anti-PD-1 (αPD-1) and anti-PD-L1 (αPD-L1)) in two different models (4T1 and TC1). The response rate to checkpoint therapy was expected to be enhanced by increasing the population of infiltrated T cells and modulating the immunosuppressive TME, as depicted in Fig. 8a. After local treatment with iGel, the expression level of PD-1 in T cells and PD-L1 in tumor cells was increased in the TC1 and 4T1 models (Fig. 8b, g). Upon the upregulation of PD-1 and PD-L1 after iGel treatment, αPD-1 and αPD-L1 were combined to observe any synergistic effect (Fig. 8c, h). Compared to treatment with surgery alone, intraperitoneal injection with αPD-1 or αPD-L1 alone did not result in any enhanced antitumor effects in either tumor model, while the combination of iGel with αPD-1 or αPD-L1 generated different antitumor effects according to the tumor cell type (Fig. 8d, i). The synergistic effect of iGel and αPD-L1 was observed in 4T1 tumors, resulting in 70% of mice being tumor free, while no effect was observed in TC1 tumors. In contrast, the synergistic effect of iGel and αPD-1 was dominant in TC1 tumors, while no significantly different effect was detected in 4T1 tumors. To understand the different behaviors, we analyzed the level of activated CD8^+^ T cells in the tumor and spleen and the level of IFN-γ secreted by the activated T cells after treatment with the different combinations in the 4T1 and TC1 tumor models. Consistent with the previous results, the highest levels of activated CD8^+^ T cells in the tumor and spleen (Fig. 8e, j) and IFN-γ secretion (Fig. 8f, k) were observed in 4T1 tumors treated with iGel and αPD-L1 and in TC1 tumors treated with iGel and αPD-1, respectively. Although further systematic studies should be conducted to explore the different responses to αPD-1 and αPD-L1 in different tumor models, we can speculate that they may be related to the different resistance mechanisms to checkpoint therapy depending on different TME condition^53,54^. In fact, many research groups are developing sets of biomarkers (tumor-infiltrating immune cells, PD-1/-L1 overexpression, mutational burden, tumor environmental metabolites, and genetic and epigenetic signatures) that can predict the response to various immune checkpoint inhibitors, and those parallel studies will be helpful to reveal the underlying mechanism of the different responses to αPD-1 and αPD-L1 in different tumors^55^. Furthermore, it should be emphasized that local treatment with iGel reversed checkpoint therapy-non-responding tumors to checkpoint therapy-responding tumors with a synergistic antitumor effect.

## Discussion

We suggested a syringeable multidomain nanogel as a synthetic immune niche that could prime the antigen-specific immune response and modulate immunosuppressive cells in the TME. iGel could reshape tumor-induced immunosuppressive TMEs via the spatiotemporal release of immunomodulatory drugs targeting MDSCs or TAMs, as well as immunostimulants, which could activate recruited APCs and generate antigen-specific T cells. Local immunomodulation of immunosuppressive TME in the post surgical bed by iGel could induce a systemic and memory antitumor immune response, which inhibited tumor recurrence at primary and secondary tumor sites and metastasis into various organs, such as lung, intestine, liver and spleen, while minimizing systemic toxicity. With the modulation of immunosuppressive MDSCs and TAMs in the TME, the increased levels of CD8^+^ T cells and IFN-γ secretion in the tumor and spleen mediated by iGel could also change checkpoint therapy non-responding tumors into checkpoint therapy-responding tumors.

In this research, we designed a local synthetic immune niche with a clinically translatable liposome-based formulation. We proposed a nanotechnology-based material design strategy to overcome the limitations of current nano-sized single liposome technology (low loading capacity and stability and short period of drug release) by developing non-concentric MNDVs. The unique structure of MNDVs enabled hydrophilic and hydrophobic immunomodulatory drugs to be loaded simultaneously and released for extended period, which was impossible by conventional liposomes or hydrogels. In fact, the pharmaceutical grade of multivesicular liposome was already developed and clinically approved for pain-management drug formulations (Depocyt®, DepoDur^TM^, Exparel®, and NOCITA®)^40^. However, such conventional multivesicular structure where micro-sized aqueous compartments were interconnected by triolein only was not so stable and resulted in heterogeneous size distributions (Supplementary Fig. 1). In this research, we suggested that the structural stability of nano-sized multidomain vesicles as well as the release profile of loaded drugs could be improved by the introduction of other oil components (squalene and oleic acid) as biological glue. Furthermore, the introduction of oleic acid was very useful to solubilize and load water-insoluble drugs into oil phase of MNDVs. It should be also emphasized that MNDVs could also be transformed into an easy-to-use, shear-force-dependent reversible gel (iGel) using CNLs. Usually, after the injection of MNDVs suspensions, it can spread into other parts and induce side effects. Owing to in situ gelling property of iGel, it would be useful to induce localized and sustained release of immunomodulatory drugs at the injection site and to prevent post surgical tumor recurrence and metastasis. Although the utility of intraoperative implantation of scaffolds to modulate immunosuppressive TME was previously suggested by our group, they still have limitation in clinical translation point of view, because the well-defined solid scaffold cannot perfectly fill the irregular size and shape of tumor surgical bed and cannot be applied into some type of inaccessible solid tumors^16,56^. In contrast, the easy-to-use and syringeable iGel could be useful for filling in the irregular cavity created after surgical removal of tumor tissues, as well as intratumoral or intranodal injection. Other research groups have also developed injectable inorganic scaffold to modulate immune cells in vivo and increase vaccine efficacy^25,57^. However, systematic studies for the toxicity profiles of inorganic materials should be preceded before their clinical translation. In fact, various nanomaterials have been tested in preclinical and clinical stages for the systemic delivery of chemotherapy drugs into surgically inaccessible tumors. However, these materials have low targeting efficiency, off-target-associated adverse effects, systemic cytokine production, and abrogation of therapeutic immune cells. In this research, iGel based on MNDVs and CNLs increased the bioavailability and allowed the use of multiple combinations of immunomodulatory drugs (gemcitabine, R837, and clodronate) that are too toxic when used systemically. Furthermore, the localized and extended release of immunomodulatory drugs from iGel could evoke systemic (abscopal) and memory immune responses, resulting in the successful treatment of inaccessible or secondary tumors. Our study also suggests that immunomodulatory small molecules that target pro-tumor inflammatory myeloid cells (MDSCs and TAMs), but have systemic severe toxicity can overcome the limitations and extend their usage by the focused and sustained release of these molecules through iGel^58^. From an in situ cancer vaccine development point of view, despite being uncertain of the dominant epitopes of a given cancer, treatment with iGel can trigger an immune response against the relevant neo-antigens or tumor-associated antigens without the need for their characterization^59,60^.

We verified the efficacy of iGel in preclinical mouse tumor models that recapitulate clinically observed tumor-induced tolerance, metastasis, and tumor heterogeneity. The iGel system is expected to be used as a versatile platform to test multiple combinations and target multiple immunosuppressive cells (MDSC, TAMs, and Tregs) or factors (IDO, VEGF, etc.) concomitantly and with high bioavailability^61^. From a clinical translation point of view, all the lipid components and drugs (gemcitabine, R837, and clodronate) used for iGel were selected based on their approval or prior use in a clinical setting, which indicated the effectiveness of iGel in humans. In the future, immunomodulatory drugs would be selected based on the tumor biopsy and immune profile analysis (i.e., lack of APCs and T cells, population of MDSCs, TAMs, and Tregs) of patients and could be loaded into iGel as a rationale for personalized immunotherapy^62^. Although the main criticism often made against local injection-based immunotherapy is about feasibility, almost every site/organ in the human body can be biopsied and injected via endoscopy and laparoscopic surgery^63^. iGel could be introduced into primary tumors located in deep tissues through image-guided intratumoral or intranodal injection^64^. iGel could be used in a wide spectrum of indications for patients with locally advanced cancers to turn inoperable tumors into operable tumors and prevent subsequent relapses through the generation of protective antitumor immunity.

In summary, we developed syringeable nanoliposome-bridged multi-nanodomain vesicle gel as a post surgical treatment that could reshape TMEs by the modulation of tumor-induced immunosuppression and the enhancement of antitumor immune priming. The reshaping of TME by the spatiotemporal delivery of chemo-immunotherapeutic drugs from iGel could also synergize cancer immunotherapy with immune checkpoint inhibitors, with minimized systemic toxicity.

## Methods

### Preparation and characterization of MNDVs

MNDVs containing gemcitabine and R837 were prepared by a two-step water-in-oil-in-water double-emulsification process. Briefly, the first step was the formation of a water-in-oil emulsion. A lipid combination solution composed of dipalmitoylphosphatidylcholine (DOPC, 16 mg), 1,2-dipalmitoylphosphatidylglycerol (DPPG, 2 mg), triolein (4 mg), cholesterol (8 mg), squalene (4 mg), and R837 (2 mg in 6 mg oleic acid) was dissolved in 1 mL chloroform. Then, the lipid solution was emulsified with an equal volume of an aqueous solution (5% sucrose and 7.5% glucose) containing 10 mg gemcitabine using a model VCX 750 microtip probe sonicator (Sonics & Materials) for 2 min on ice to produce a water-in-oil emulsion. Subsequent emulsification with a second aqueous solution (5 mL 40 mM lysine and 7.5% glucose) using a Power Gen 1000 homogenizer (Thermo Fisher Scientific) yielded a water-in-oil-in-water double emulsion. Then, the chloroform was removed by rotary evaporation at room temperature for 30 min to form MNDVs. The resulting MNDVs were washed to remove unencapsulated drug, harvested by centrifugation for 10 min at 600 × *g* and resuspended in the second aqueous solution. The final products were stored at 4 °C for further use. For preparation of C-liposomes, the lipid components (same as those of MNDVs) and drug were dissolved in chloroform. The solution was then dried to a thin-film using a rotary evaporator. Lipid films were rehydrated in phosphate-buffered saline (PBS) at 60 °C for 30 min, with continuous stirring at 300 rpm. The resulting solution was sonicated using a microtip probe sonicator for 1 min on ice.

The gemcitabine and R837 encapsulation amounts were determined by ultraviolet-visible (UV–Vis) spectrometry at wavelengths of 265 and 245 nm, respectively. The MNDV size was obtained using a DeltaVision™ PD system (GE Life Sciences) and was quantitatively analyzed using ImageJ software (NIH). Fluorescence images of MNDVs loaded with FITC-OVA and DID were obtained using the following filter set: FITC, excitation 490/20, emission 525/36; and Cy5, excitation 632/22, emission 679/34 (Omega Optical). The internal structure of MNDVs was analyzed by cryogenic TEM (JEOL 2100, JEOL Ltd.) and SEM (TESCAN MIRA3 LMU).

### Preparation of clodronate-CNLs

To prepare clodronate-CNLs, we dissolved dioleoylphosphatidylethanolamine (DOPE, 0.006 m mole) and N-[1-(2,3-dioleyloxy)propyl]-N,N,N-trimethylammonium chloride (DOTMA, 0.006 m mole) in 1 mL chloroform and generated a thin film by rotary evaporation at 25 °C for 30 min. The thin film of the lipid mixture was resolubilized in deionized water containing 1 mg clodronate. The resulting solution was sonicated for 1 min and then reacted for 2 h to form a stable complex. Nonencapsulated clodronate was removed using a centrifuge filter (Spectra/Por tube, 50 kDa MW cutoff). The amount of encapsulated clodronate was then determined. Briefly, the lyophilizing clodronate-CNL was redissolved in the solution containing 1.5 mM nitric acid and 1.5 mM Copper (II) sulfate (pH 2.8). The clodronate encapsulation amounts were determined by UV–Vis spectrometry at wavelengths of 236 nm. The size distribution and zeta potential of clodronate-CNLs were analyzed by dynamic light scattering using an ELS-Z electrophoretic light scattering photometer (Otsuka Electronics Co.).

### Mice and cell lines

BALB/c and C57BL/6 mice (female, 6-weeks-old) were purchased from Orient Bio and maintained under pathogen-free conditions. The animal study was reviewed and approved by the Institutional Animal Care and Use Committee (IACUC) of Sungkyunkwan University School of Medicine, which is accredited by the Association for Assessment and Accreditation of Laboratory Animal Care International (AAALAC International) and abides by the Institute of Laboratory Animal Resources (ILAR) guide. Murine 4T1 (triple-negative breast cancer), murine TC1 (cervical cancer), and human THP-1 cells (American Type Culture Collection, ATCC) were cultured in RPMI medium (Thermo Fisher Scientific). Human C33a cervical cancer cells (ATCC) were cultured in Dulbecco’s Modified Eagle Medium (DMEM) medium (Thermo Fisher Scientific). The cell lines used in this study are not listed in the database of misidentified cell lines. The cell lines used in our study were tested to be free of mycoplasma contamination.

### In vitro effects of gemcitabine

Gemcitabine induced the apoptosis of MDSCs, 4T1, C33a, T cells and BMDCs in vitro. The spleen was harvested from mice bearing 28-day established 4T1 subcutaneous tumors; next, the spleen was homogenized and treated with red blood cell lysis buffer. Splenocytes were resuspended in MACS buffer (PBS, 0.5% BSA, 2 mM EDTA). MDSCs were isolated via negative selection using an MDSC Isolation Kit (Miltenyi Biotec). Purified MDSCs, 4T1, C33a, T cells and BMDCs were seeded on 24-well plates at a density of 1 × 10^6^ cells/well, followed by the placement of permeable membranes (12-mm diameter, polycarbonate membrane, 3-µm pore size, Corning Inc.). Then, 50 µL gemcitabine, gemcitabine-loaded MNDVs, blank MNDVs, and PBS was placed on the membrane and incubated with the cells for 24 h. The induction of apoptosis was analyzed using a FITC Annexin-V Apoptosis Detection kit (BD Biosciences) according to the manufacturer’s instructions and a BD FACSCanto™ II flow cytometer.

Gemcitabine-loaded MNDVs induced ICD in vitro. CALR expression on cultured 4T1 and TC1 cells was examined using a BD FACSCanto™ II flow cytometer 4 h after treatment with gemcitabine, gemcitabine-loaded MNDVs, blank MNDVs, and PBS. Fluorescence images were obtained using a DeltaVision™ PD system (GE Life Sciences). 4T1 cells were fixed in 4% (w/v) paraformaldehyde for 20 min at room temperature and stained with Hoechst 33342 solution (2 µg mL^−1^, Thermo Fisher Scientific) in PBS for 10 min. HMGB1 expression was examined 24 h following the treatment by ELISA (ARG81310, Arigo Biolaboratories).

### In vitro activation in response to cancer vaccine treatment

Tumor cells were incubated with gemcitabine-loaded MNDVs (5 µg mL^−1^) overnight and then the supernatants were collected. BMDCs or macrophage-like THP-1 cells were seeded on 24-well plates at a density of 1 × 10^6^ cells/well, followed by the placement of permeable membranes (12-mm diameter, polycarbonate membrane, 3-µm pore size, Corning Inc.). Then, R837-loaded MNDVs (1 µg mL^−1^), media conditioned by gemcitabine-loaded MNDVs-treated tumor cells, or both were placed on the membrane and incubated with the cells for 24 h. The culture supernatants were collected, and the levels of secreted IL-6 and TNF-α were analyzed by ELISA (BD Biosciences) according to the manufacturer’s instructions. BMDCs were stained with APC-conjugated anti-CD11c (clone: N418, catalog number: 117309, dilution: 1:100) and FITC-conjugated anti-CD40 (clone: 3/23, catalog number: 124607, dilution: 1:250) or FITC-conjugated anti-CD80 (clone: 16–10A1, catalog number: 104705, dilution: 1:250) antibodies for analysis by flow cytometry.

In vitro cell viability test of clodronate-CNLs BMDMs, 4T1, and M2 macrophage-like THP-1 cells were seeded into 96-well plates at a density of 1 × 10^4^ cells/well and cultured for 12 h. The culture media were then replaced with fresh culture media containing free clodronate, CNLs, and clodronate-CNLs at different drug concentrations. After 24 h of incubation, cell viability was measured using the colorimetric MTS test (CellTiter 96^**®**^).

### In vivo fluorescence imaging of iGel retention

MNDVs were labeled with IR dye 800 and fabricated iGel was subcutaneously injected into the right flank of mice. Near infrared images (0.2 s of exposure) of mice were acquired using a diode laser at 785 nm with 500 mW as an excitation light source and an 835/45-nm bandpass emission filter. All images were processed using Simple PCI software. Fluorescence signals were quantitatively analyzed using ImageJ software.

### Toxicity analysis

The body weight of mice was measured, and blood was collected from mice at the indicated time intervals after treatment. Serum was separated from blood cells by centrifugation at 10,000 × *g* for 10 min at 4 °C. Serum ALT, AST, and BUN levels were measured using a transaminase assay kit. The serum IL-6 level was measured by ELISA according to the manufacturer’s instructions. The liver, lungs, and kidneys were also excised from mice 1 day after treatment; the samples were embedded in optimal cutting temperature compound, sectioned at a thickness of 5 μm, stained with haematoxylin and eosin and observed for signs of toxicity by light microscopy.

### Surgical procedure and implantation of iGel

On days 14 and 12 post inoculation of 1 × 10^6^ 4T1 and TC1 cells, respectively, when the primary tumors were ~300 mm^3^ in size, the tumors were partially debulked. The mice were anaesthetized with 2,2,2-tribromoethanol (Sigma-Aldrich), the surgical area was sprayed with 70% ethanol, and ~90% of the tumor was removed, leaving 10% residual tissue behind. iGel was implanted directly beside the residual tumor.

### Analysis of infiltrating immune cells

Seven days after the surgical procedure, tumors, spleen, and tumor-draining lymph nodes were harvested. The tumors and lymph nodes were cut into small pieces and resuspended in collagenase D in DMEM (1 mg mL^−1^). The solutions were incubated for 1 h at 37 °C on a shaker and then filtered through a 70-µm Falcon cell strainer. The supernatant from the digested tumor tissues was collected, centrifuged at 490 × *g* for 5 min, and resuspended. The spleen was mechanically dissociated and resuspended in DMEM. The suspension was filtered through a 70-µm Falcon cell strainer, centrifuged, and resuspended. Erythrocytes were lysed with red blood cell lysis buffer for 5 min at 37 °C.

Cell suspensions were prepared as described above and then stained with the following antibodies: APC-conjugated anti-CD11b (clone: M1/70, catalog number: 101212, dilution: 1:100), and PE-conjugated anti-Gr1 (clone: RB6–8C5, catalog number: 108408, dilution: 1:100) antibodies for MDSCs; FITC-conjugated anti-CD3 (clone: 17A2, catalog: 100204, dilution: 1:250), PE-conjugated anti-CD4 (clone: RM4–4, catalog number: 116006, dilution: 1:100), and APC-conjugated anti-CD8 (clone: 53–6.7, catalog number: 100712, dilution: 1:100) antibodies for T cells; FITC-conjugated anti-CD206 (clone: C068C2, catalog number: 141704, dilution: 1:250), and PE-conjugated F4/80 (clone: BM8, catalog number: 123110, dilution: 1:100) for M2 macrophages; FITC-conjugated anti-CD3 and APC-conjugated anti-CD335 (clone: 29A1.4, catalog number: 137602, dilution: 1:250) for NK cells. Unless otherwise indicated, all antibodies were purchased from BD Biosciences and BioLegend. The cells were then washed twice and analyzed using an Accuri™ flow cytometer (Supplementary Fig. 21).

### Analysis of the antigen-specific response

The ex vivo antitumor response was analyzed. Cell suspensions prepared from the spleen and lymph nodes isolated from mice were washed twice with PBS. Lymphocytes were cultured in 12-well plates at 1 × 10^6^ cells/mL and restimulated with 4T1 or TC1 cell lysate (80 µg mL^−1^). After 72 h of culture, the supernatants were harvested and stored at −20 °C until assayed with IL-2, TNF-α, and IFN-γ ELISA kits (BD Biosciences).

### Systemic antitumor immunity

BALB/c mice were inoculated with 1 × 10^6^ 4T1 tumor cells in the right flank (primary tumor). On day 14 (post inoculation), primary tumors were partially debulked, and the left flank was inoculated with cells for secondary tumor formation. After 14 days, primary and secondary tumors were harvested and analyzed regarding T-cell infiltration using a BD FACSCelesta™ flow cytometer.

### In vivo antibody depletion

Monoclonal GK1.5 antibodies (catalog number: BE0003–1) were used for CD4 depletion, mAb 2.43 antibodies (catalog number: BE0061) were used for CD8^+^ T-cell depletion, and anti-mouse CD49b antibodies (catalog number: 146002) were used for NK cell depletion. The depletion was started 1 day before the surgery, and the injections were continued during therapy.

### Metastatic analysis

On day 21 after tumor resection, mice were sacrificed, and organs (intestine, liver, and spleen) were excised. Metastatic nodules were counted by visual observation. Lung metastasis was quantified by an intratracheal injection of India ink. The lungs were then extracted and immersed in Fekete’s solution for destaining (100 mL 70% ethanol, 10 mL 4% formaldehyde, and 5 mL 100% glacial acetic acid); metastatic nodules were counted by visual observation.

MRI was performed using a Biospec 47/40 USR AV III 4.7-T scanner (Bruker). T_2_-weighted MRI scans were obtained using the following parameters: rapid acquisition with relaxation enhancement sequence; TE/TR = 36/3600 ms; matrix size, 192 × 192; field of view, 3 × 3 cm; slice thickness, 1 mm. Mice were placed in the prone position. All images were acquired in the coronal and axial orientations.

### Combination of iGel with immune checkpoint blockage

Three days after surgery, αPD-1 (clone: RMP1–14, catalog number: BE0146) and αPD-L1 (clone: 10 F.9G2, catalog number: BE0101) antibodies (Bio X Cell), each at a dose of 200 µg per mouse, were administered intraperitoneally five times every 2 days.

### Statistical analysis

All results are presented as the mean ± standard deviation. When normality assumptions were met (Shapiro–Wilk normality test), two-tailed unpaired Student’s *t-*test was used for comparing two groups of data and one-way ANOVA with corresponding Tukey’s multiple comparison was used for multiple groups of data. When normality assumptions were not met, nonparametric statistical tests were performed. Mann–Whitney or Kruskal–Wallis test with Dunn’s multiple comparison post hoc test was performed in the comparison of two or more groups. Significance is indicated by *P* < 0.05 (*P < 0.05, **P < 0.01, ***P < 0.001). Differences in survival were determined for each group by the Kaplan–Meier method, and the overall *P-*value was calculated by the log-rank test. GraphPad Prism 7 (GraphPad software) and Excel 2016 (Microsoft Corporation) were used for all statistical analyses. The numbers of animals included in the study are discussed in each figure legend.

### Reporting summary

Further information on research design is available in the Nature Research Reporting Summary linked to this article.

## Supplementary information

Supplementary Information

Reporting Summary

Source data

## Data Availability

The source data underlying Figs. 1c–e, 2b–c, 3a–g, 4b–d, 5b–d, 6b–e, g, 7a, d–g, 8b, e–g, j, k and Supplementary Figs. 1a, 4b, 5b, 9a–c, 10a, 12a, b, 13a, c–e, 14c–e, 17a, b, 18a–c, and 19b are provided as a Source Data file. All the other data supporting the findings of this study are available within the article and its supplementary information files and from the corresponding author upon reasonable request. A reporting summary for this article is available as a Supplementary Information file.
