# Supplementary material for: Syringeable immunotherapeutic nanogel reshapes tumor microenvironment and prevents tumor metastasis and recurrence
Source: Nat Commun. 2019 Aug 20;10:3745. doi: 10.1038/s41467-019-11730-8 (PMC6702226; doi:10.1038/s41467-019-11730-8)
Supplement: Supplementary file 1 — Supplementary Information [file 41467_2019_11730_MOESM1_ESM.pdf]

Supplementary Information

Syringeable immunotherapeutic nanogel reshapes tumor microenvironment and prevents tumor metastasis and recurrence

Song et al.

Supplementary Methods

Evaluation of the stability of MNDVs and iGel

MNDVs and the mixture of MNDVs and CNLs (10:3 ratio w/w) were maintained at 4°C and 37°C for the indicated time periods. The solutions were sampled to observe the clustering and morphology of the particles using a DeltaVision™ PD system (GE Life Sciences).

In vitro release of gemcitabine and R837 from MNDVs and iGel

MNDVs were loaded with gemcitabine or R837, placed in dialysis tubing (MW cutoff, 10 kDa) and dialysed against 25 mL PBS pH 7.4 in an incubator shaker at 37°C and 90 rpm. At selected time intervals, the buffer solution outside the dialysis tubing was removed and replaced with fresh buffer solution. The gemcitabine and R837 concentrations were determined by UV–Vis analysis at wavelengths of 265 and 245 nm, respectively.

In vitro release of CNLs from iGel

FITC-conjugated DOPE was mixed with DOPE and DOTMA to formulate CNLs. FITC-labelled nanoliposomes were mixed with MNDVs and then placed into Transwell inserts (12-mm diameter, polycarbonate membrane, 3-µm pore size, Corning Inc.) that had previously been inserted into 24-well plates containing 1 mL PBS. At selected time intervals, the release medium was collected and replaced with fresh media. The amount of nanoliposome release was determined by measuring the UV absorbance at 495 nm.

Rheological characterization

The rheological behavior of self-assembled iGel was characterized using an ARES-G2 rheometer (TA Instruments). All measurements were performed at 25°C using a flat steel plate geometry (25 mm in diameter) with a gap distance of 1000 µm. First, the viscosity of iGel

compared to that of MNDVs alone was evaluated with an increasing rate of shear force by the stepped flow test (0.1-100 s^{-1}, 1 Hz frequency). Next, the recoverability of the gel after network destruction was assessed. The storage modulus (G') and loss modulus (G'') of self-assembled gels were measured by oscillatory time sweep experiments (3 min, 0.2% strain, 1 Hz frequency) before severe destruction of the gel network (1 min, 500% strain, 1 Hz frequency); then, the former oscillatory time sweep mode was restored (2 min, 0.2% strain, 1 Hz frequency).

Generation of BMDCs and BMDMs from mice

Both femurs and tibiae were collected, and muscle attachments were carefully removed. Intact bones were disinfected by soaking in 70% ethanol for 1 min and were then washed with PBS. Both bone ends were cut, and the marrow was flushed with RPMI and DMEM using a syringe equipped with a 26-gauge needle for BMDCs and BMDMs, respectively. After one wash (490 × *g*, 5 min) in media, the red blood cells were lysed with red blood cell lysis buffer (Sigma-Aldrich).

For BMDCs, bone marrow cells (5×10^5 cells) were collected and cultured in sterile 100-mm Petri dishes containing RPMI medium (10 mL) supplemented with 10% heat-inactivated FBS, penicillin, streptomycin, and mouse recombinant granulocyte macrophage colony-stimulating factor (GM-CSF, 20 ng mL^{-1}; PeproTech). For BMDMs, bone marrow cells (5×10^5 cells) were collected and cultured in sterile 100-mm Petri dishes containing DMEM (10 mL) supplemented with 20% heat-inactivated FBS, penicillin, streptomycin, and mouse recombinant M-CSF (20 ng mL^{-1}; PeproTech). After 7 days, BMDCs and BMDMs were harvested, washed, and used for further *in vitro* experiments.

Generation of human M2 macrophage-like THP-1 cells

Human monocytic THP-1 cells were cultured in RPMI containing 10 % of heat inactivated FBS, penicillin and streptomycin. THP-1 monocytes are differentiated into macrophages by 24 h incubation with 100 ng mL^{-1} of phorbol 12-myristate 13-acetate followed by 24 h incubation in RPMI medium. Macrophages were polarized into M2 phenotype by incubation with 20 ng mL^{-1} of IL-4 for 48 h.

***In vivo* cytokine analysis**

Tumor tissue (~100 mg) was excised and homogenized in protein extraction buffer containing protease inhibitor (1 mL) to determine TNF-α, IL-6, IFN-γ and IFN-α concentrations in recurring tumors. Cytokine concentrations in the tissue were then measured by ELISA according to the manufacturer's instructions and determined as pg/mg protein for 4T1 and pg/mg tumor for TC1 model.

***In situ* immunofluorescence of recurring tumor**

Recurring tumors were dissected 7 days after surgical resection of the primary tumor and then embedded in Tissue-Tek OCT compound (Sakura) and frozen. Cryosections (10 μm) were prepared using a model CM1850 cryostat (Leica Microsystems) and transferred to glass slides. The sections were fixed with 4% formaldehyde for 5 min, dried, and frozen at -20°C until use. After washing, the slides were stained with anti-mouse CD8α (ab217344, Abcam) overnight at 4°C. The slides were then stained with FITC-conjugated anti-rabbit IgG secondary antibodies (ab150077, Abcam) for 1 h at room temperature. The slides were washed twice with PBS and then treated with 2 μg mL^{-1} Hoechst 33342 in PBS for 10 min. After the final wash, the slides were mounted in 50% glycerol and examined using a DeltaVision™ PD system (GE Life Sciences).

Supplementary Figures

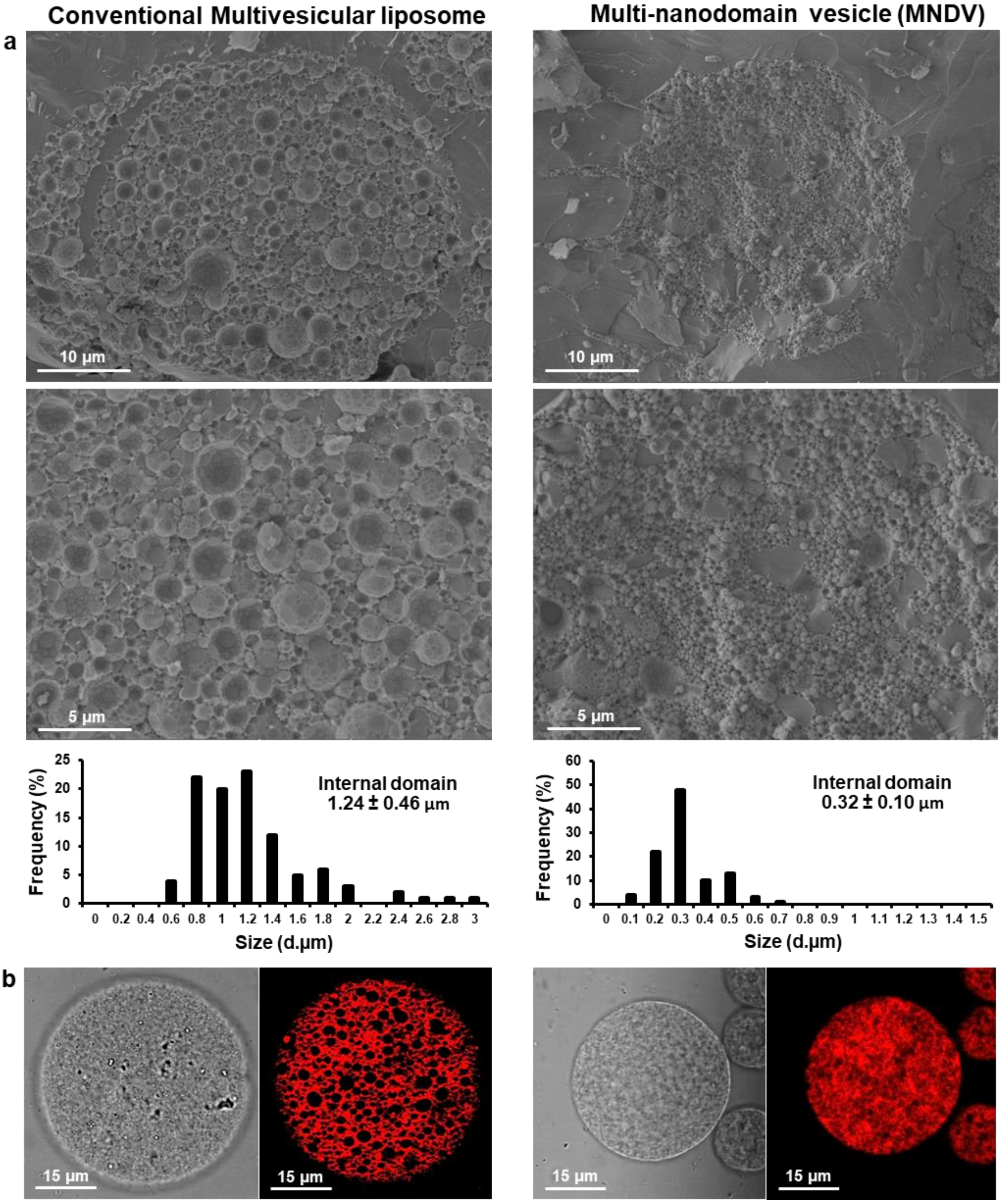

Supplementary Fig. 1. The structure of conventional multivesicular liposomes and multi-nanodomain vesicles. **(a)** The structure was observed by Cryo SEM. Summary of ImageJ analysis showing the mean diameter (n = 100). **(b)** The structure was observed by confocal microscopy labelled lipid component with DID. Source data are provided as a Source Data file.

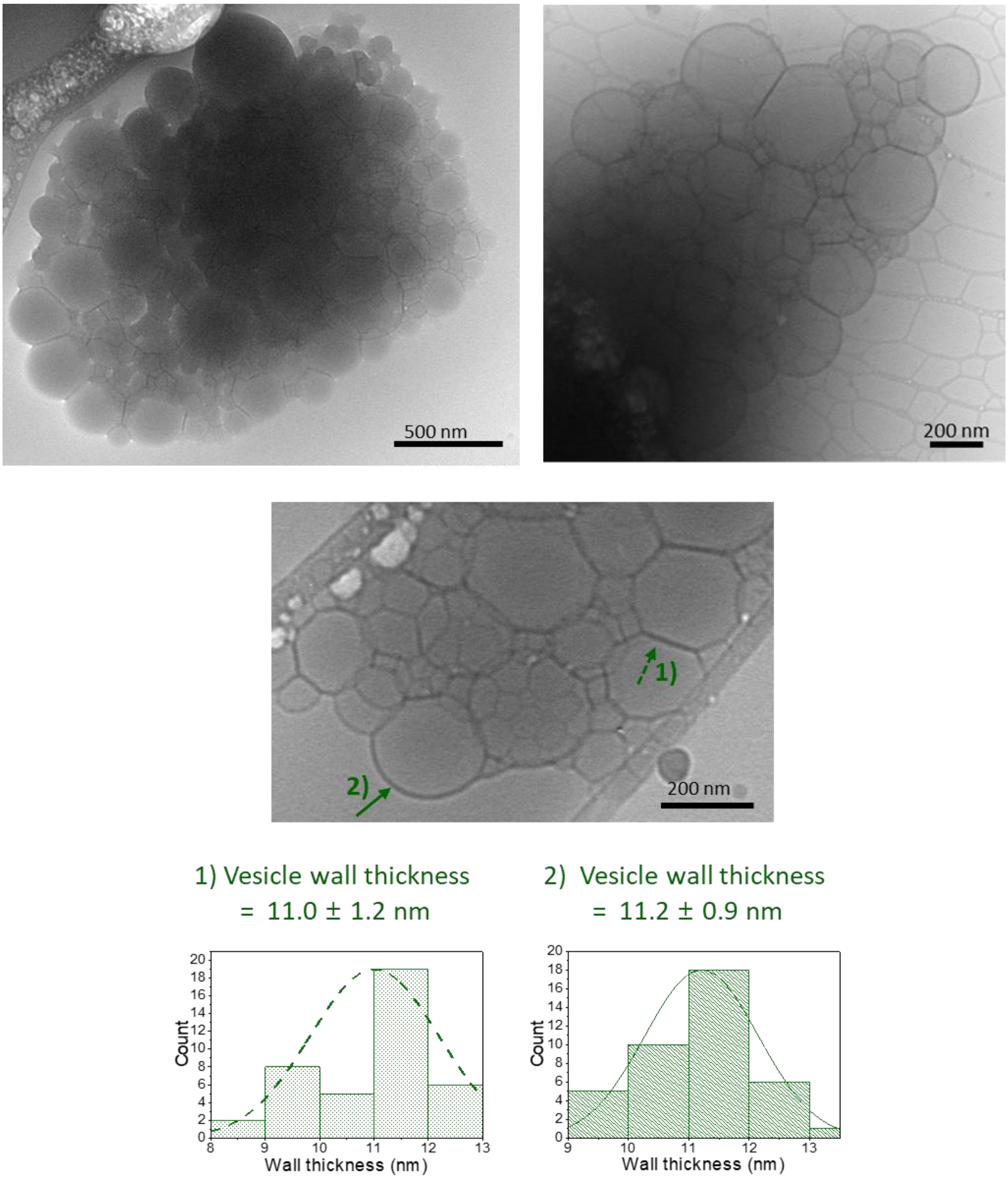

Supplementary Fig. 2. Transmission electron cryomicrographs of the internal structure of MNDVs which are composed of multiple non-concentric aqueous compartments surrounded by a network of lipid membranes. To get a high resolution image for the internal structure, small sized MNDV was selected for Cryo-TEM measurements.

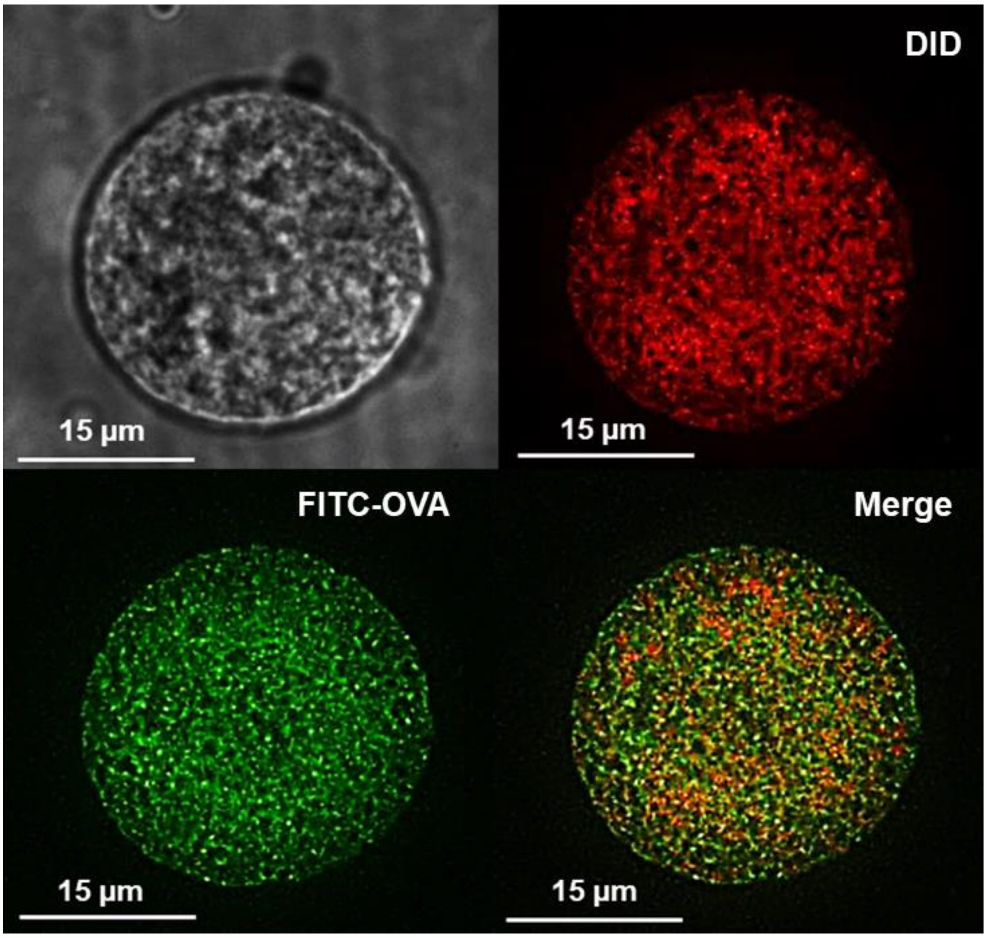

Supplementary Fig. 3. Fluorescent images of MNDVs showing the hydrophilic (FITC-OVA) and hydrophobic (DID) compartments were obtained using DeltaVision™ PD.

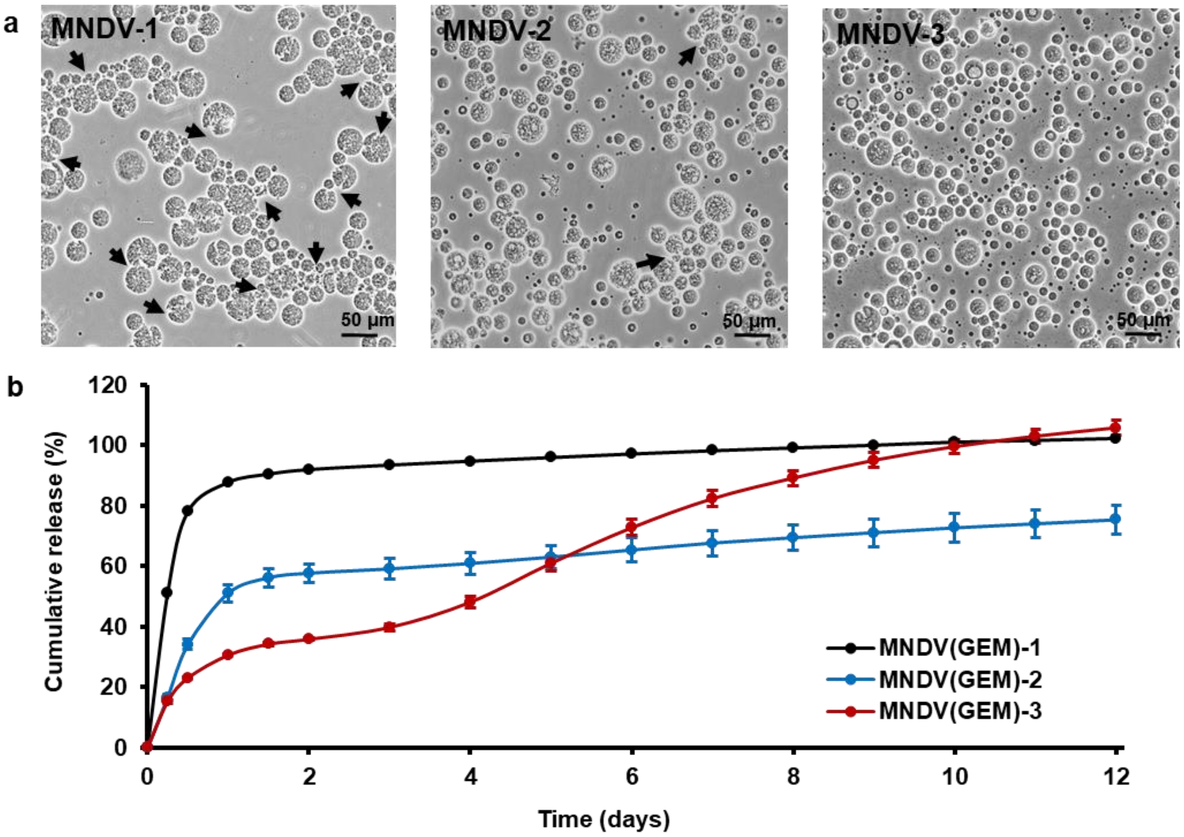

Supplementary Fig. 4. Characterization of different MNDV formulations. **(a)** Photograph of MNDVs observed by optical microscopy with different oil components in MNDV formulations: MNDVs containing triolein (MNDV-1), MNDVs containing triolein and squalene (MNDV-2), MNDVs containing triolein, squalene and oleic acid (MNDV-3). Arrows indicate the unstable particles. **(b)** Release of gemcitabine from MNDVs with different oil components. Data are presented as the mean ± SD (n = 3). Source data are provided as a Source Data file.

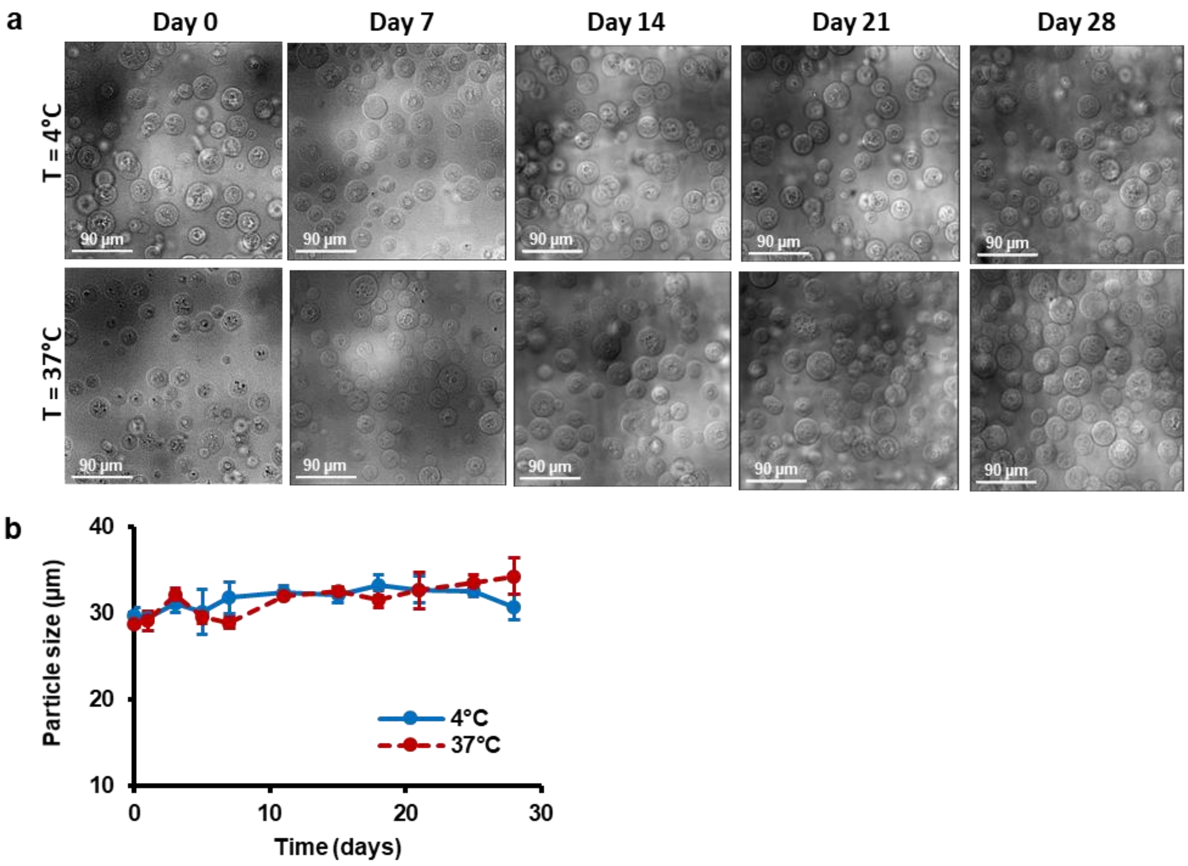

Supplementary Fig. 5. Stability of MNDV was maintained at 4°C and 37°C. **(a)** MNDV images were obtained using DeltaVision™ PD in triplicate, and **(b)** particle size was determined via ImageJ image analysis. Diameter was expressed as the mean of random samples (n = 25) from each image. Source data are provided as a Source Data file.

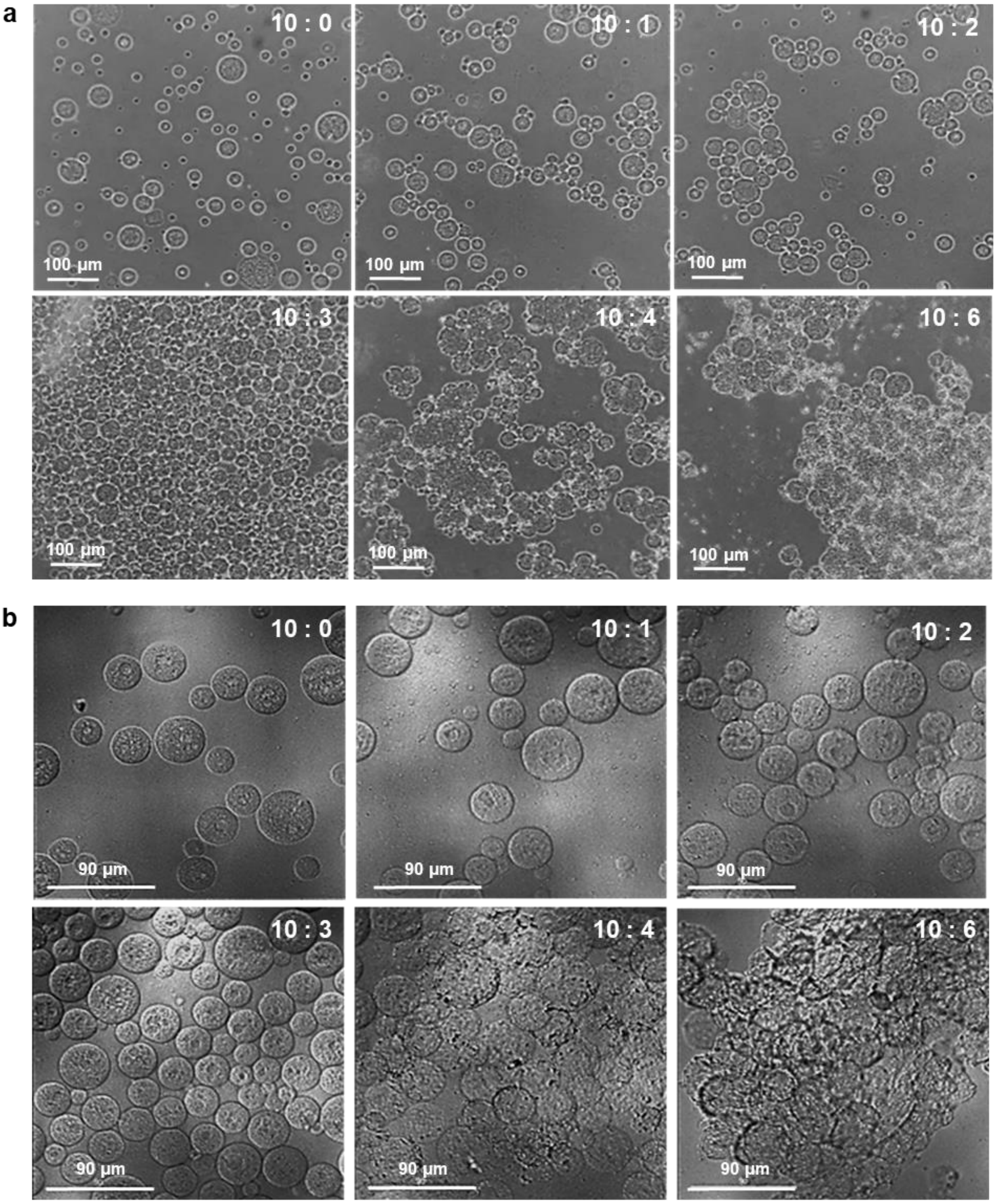

Supplementary Fig. 6. The tuning of mixing for the optimization of the clustering between MNDVs and CNLs. Morphology of the cluster with different weight ratios between MNDVs : CNLs observed by **(a)** optical microscope and **(b)** DeltaVision™ PD.

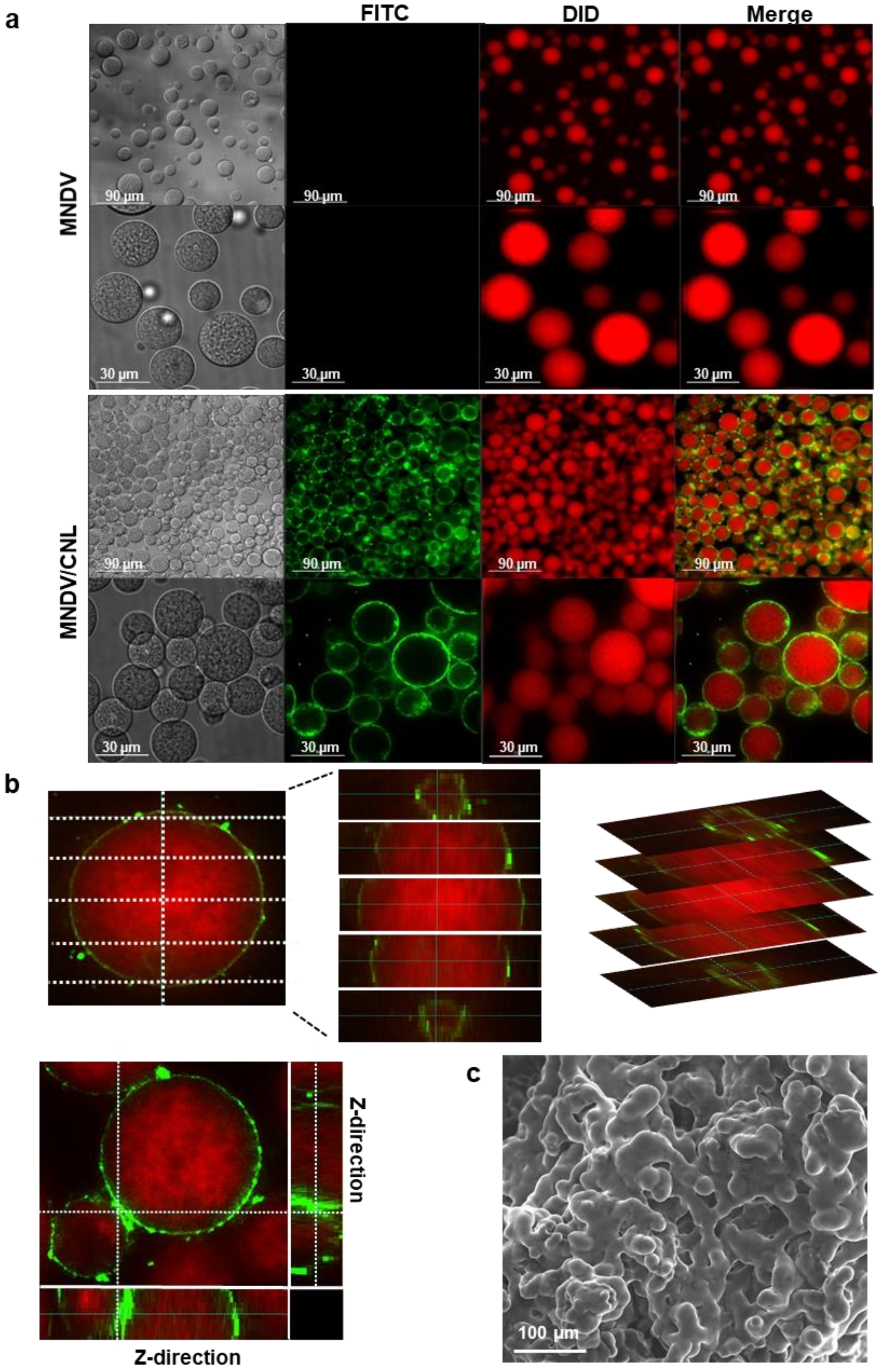

Supplementary Fig. 7. Characterization of the clustering between MNDVs and CNLs. **(a)** X-Y axis images and **(b)** Z-axis image of the cluster of DID-labelled MNDVs and FITC-labelled CNLs (10:3 ratio) were obtained using DeltaVision™ PD. **(c)** Morphology of lyophilized iGel characterized by scanning electron microscopy. A broken cross-section of the sample was sputter-coated with platinum and examined at 10 kV.

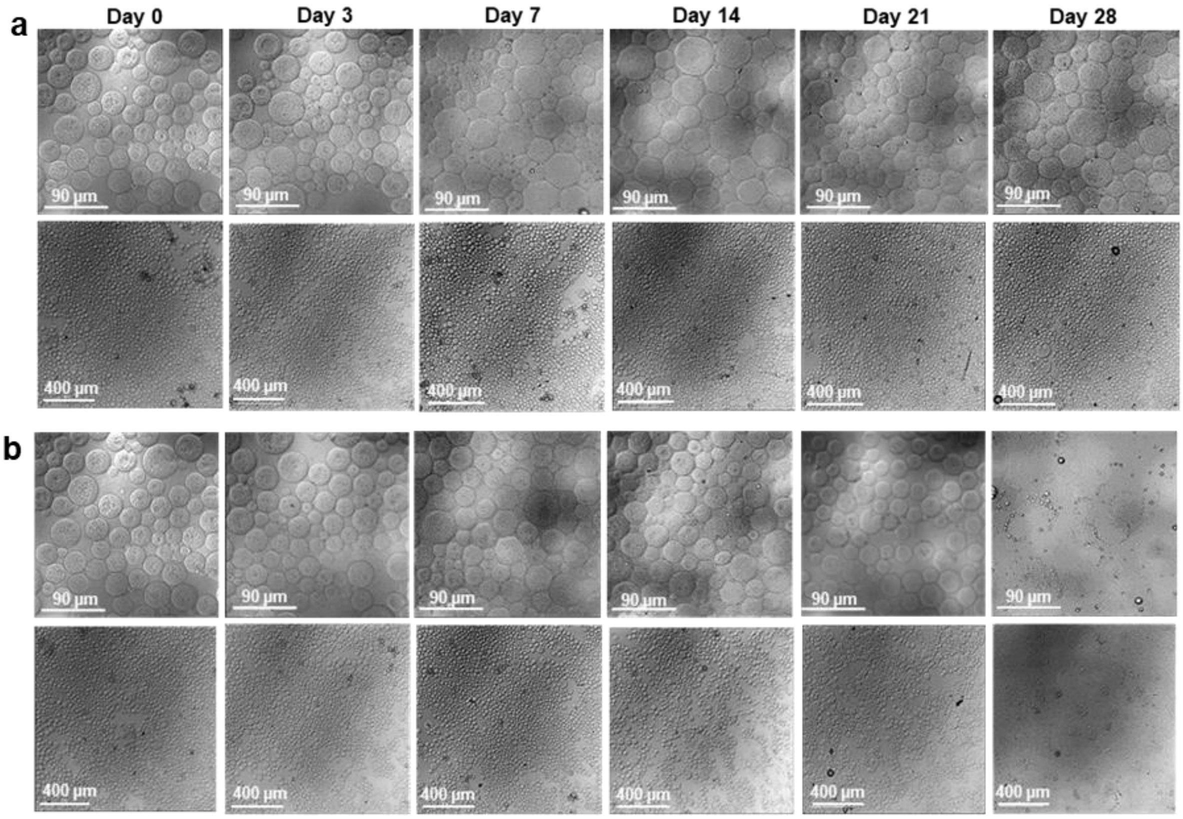

Supplementary Fig. 8. Stability of the clustering between MNDV and cationic liposome (iGel) at **(a)** 4 °C and **(b)** 37 °C which observed by DeltaVision™ PD.

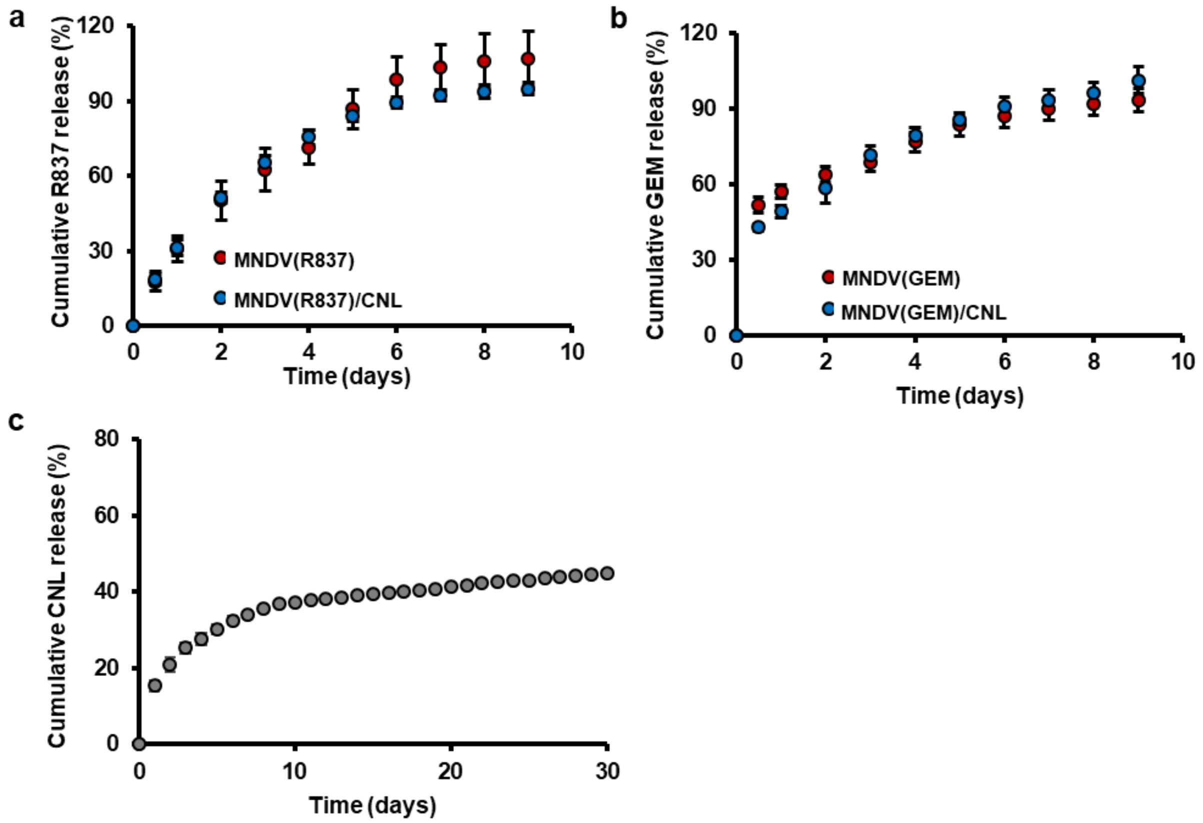

Supplementary Fig. 9. *In vitro* release of **(a)** R837 and **(b)** gemcitabine from MNDVs and MNDV/CNL gel. **(c)** *In vitro* release of FITC-labelled CNLs from the gel. Data are presented as the mean ± SD (n = 3). Source data are provided as a Source Data file.

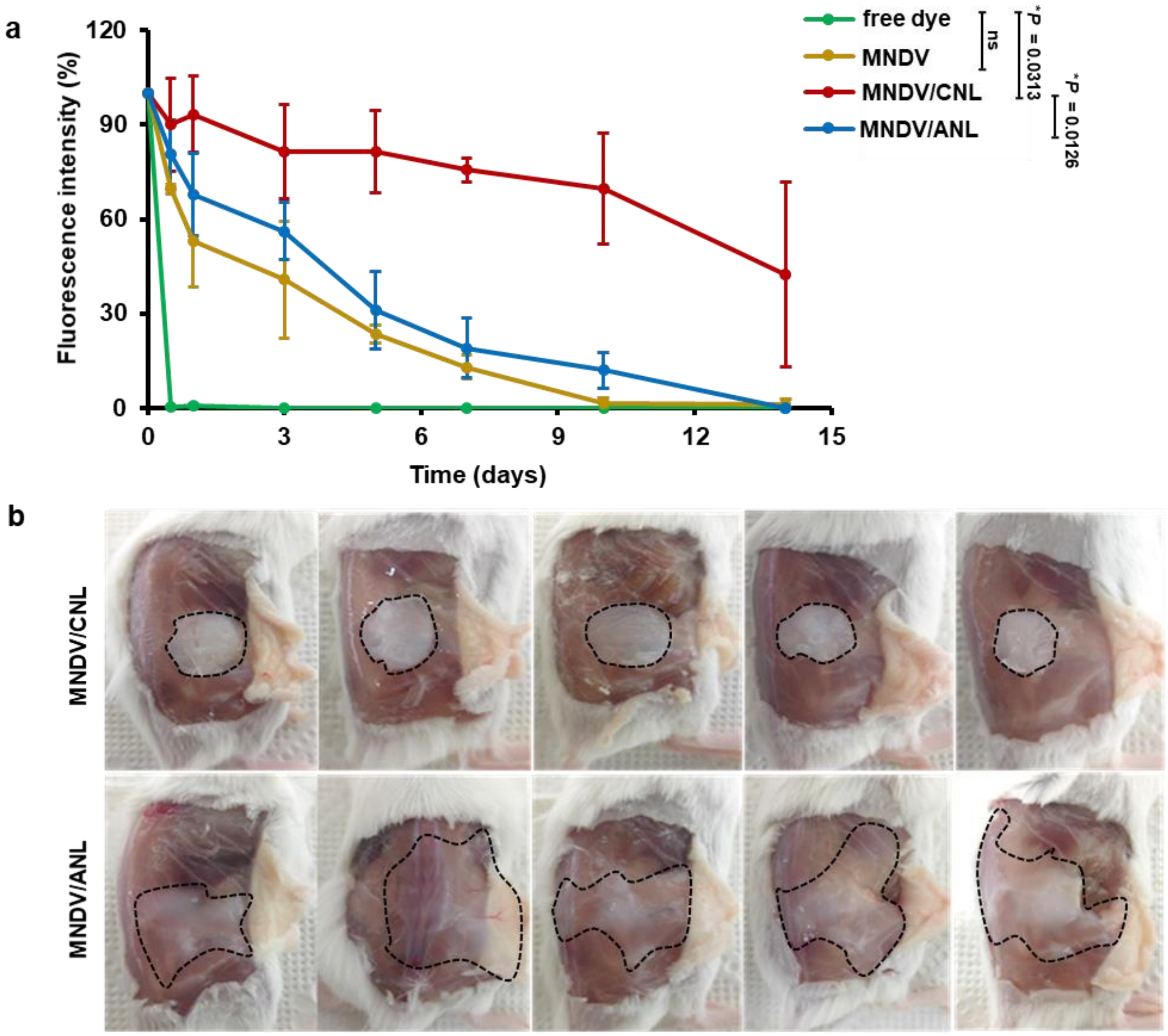

Supplementary Fig. 10. Local delivery of MNDVs/CNLs as an injectable immune niche can retain gel in the injection site. **(a)** IR dye-conjugated MNDVs and MNDVs/CNLs were injected into mice, and fluorescence imaging was performed at the indicated time points. Quantification of fluorescence imaging was analysed via ImageJ image analysis (n = 3). *P* values were analysed by one-way ANOVA and Tukey's tests (data at day 14). **(b)** Distribution of MNDVs/CNLs (upper) and MNDVs/ANLs (lower) at day 14 after injection. The dispersion and retention of samples were observed as indicated by the dotted line. Source data are provided as a Source Data file.

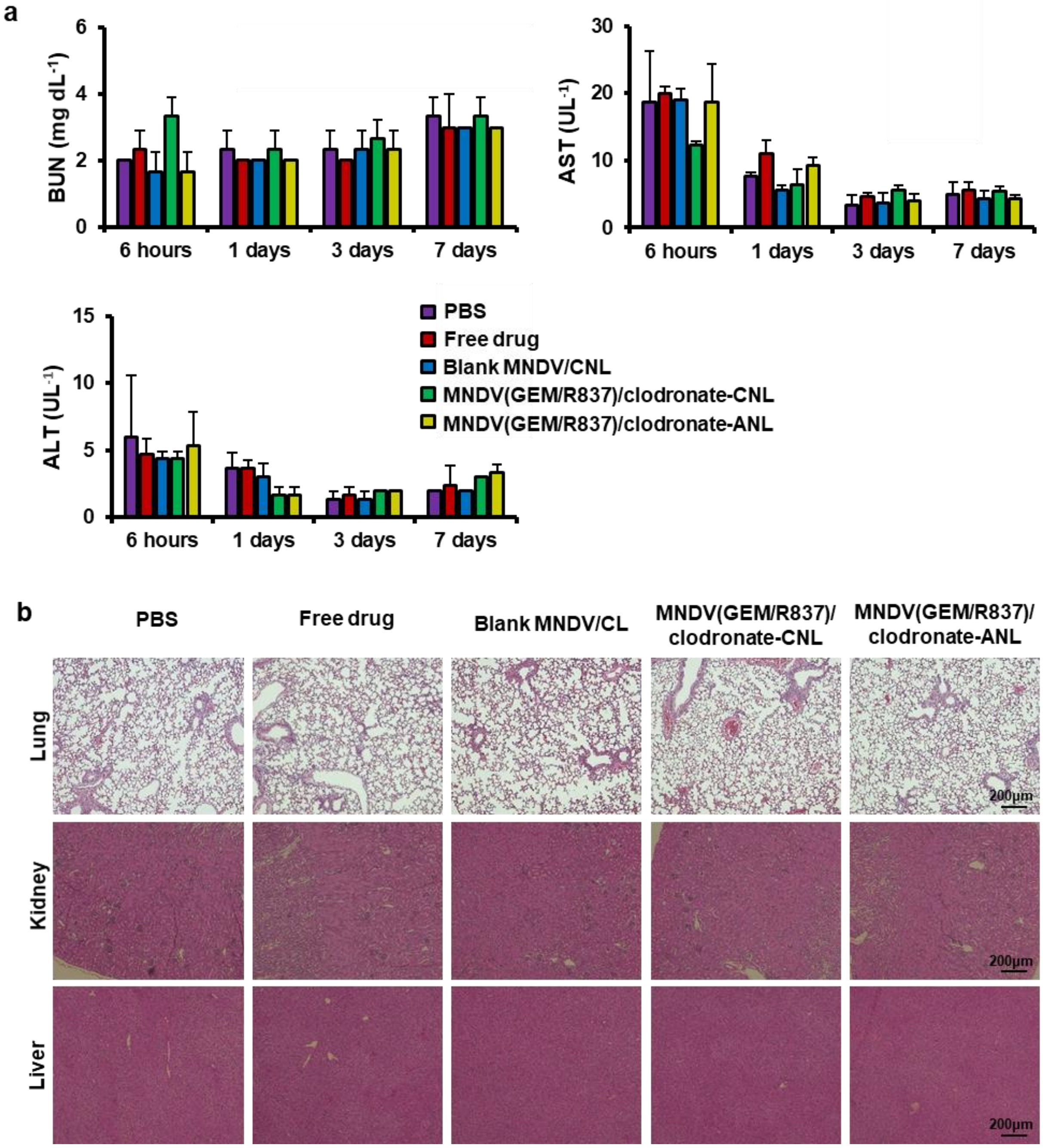

Supplementary Fig. 11. Toxicity analysis of the treatment. **(a)** The serum concentrations of BUN, AST and ALT measured at 6 h, 1, 3, and 7 days post-injection (n = 3). *P* values were analysed by Kruskal-Wallis test. No statistical difference was observed among all groups. **(b)** Haematoxylin and eosin assessment of histopathological sections of the lung, kidney and liver from treated mice at 24 h. (n = 3)

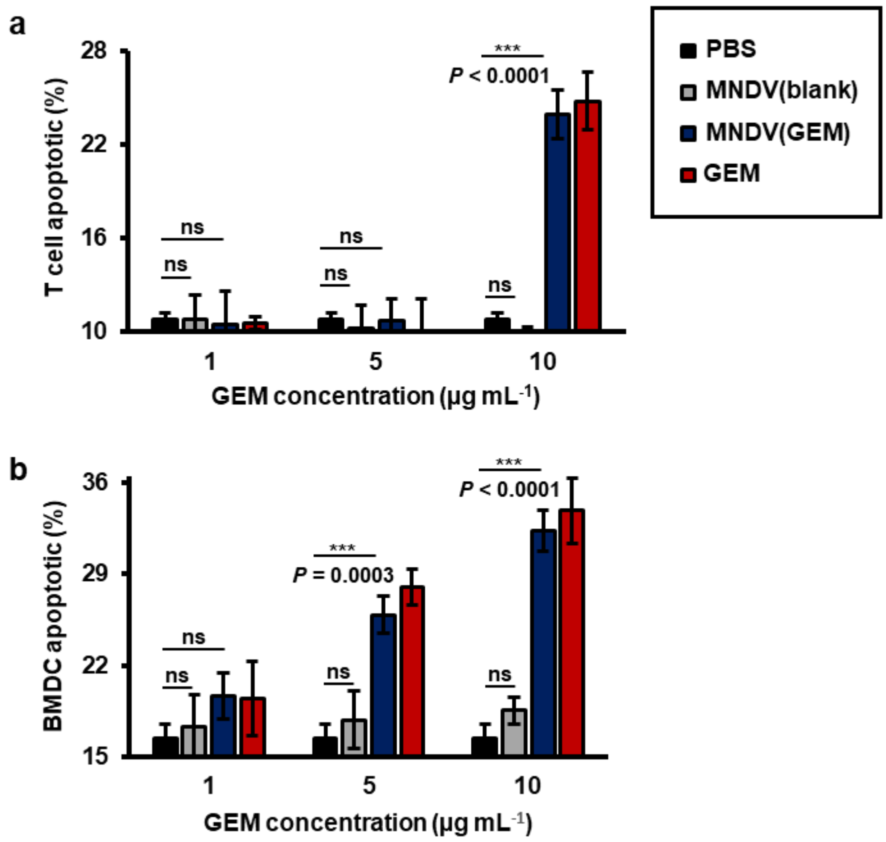

Supplementary Fig. 12. *In vitro* test of MNDV(GEM)-induced apoptosis. Percentage of apoptotic **(a)** T cells and **(b)** BMDCs (Annexin-V and PI double-positive cells). Data are presented as the mean ± SD (n = 3). *P* values were determined by one-way ANOVA and Tukey's test. Source data are provided as a Source Data file.

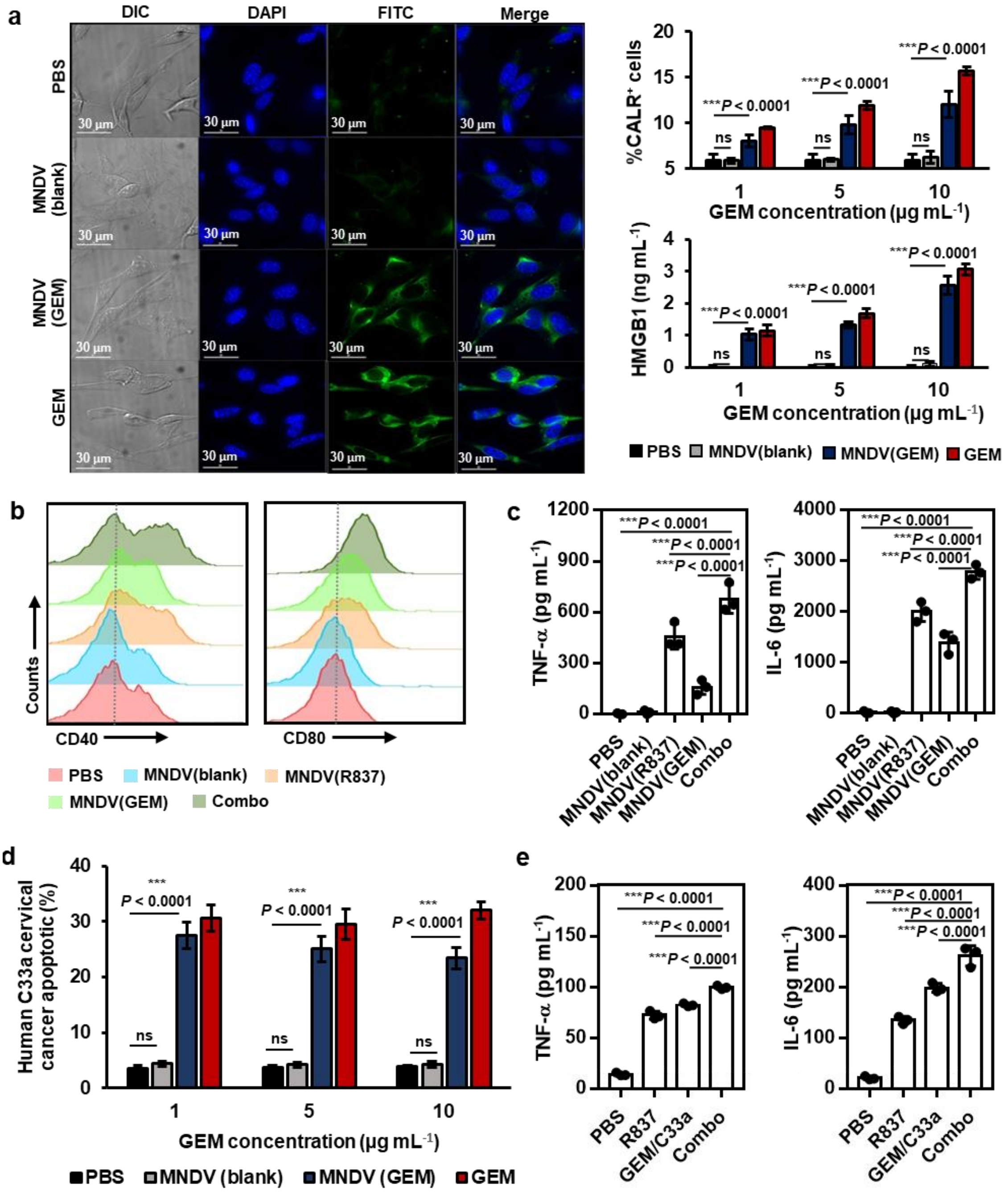

Supplementary Fig. 13. *In vitro* test of induction of *in situ* cancer vaccine in cervical cancer. **(a)** MNDV(GEM) induced immunogenic changes in TC1 cells. Confocal microscopy showing the induction of CALR in TC1 cells in presence of MNDV(GEM) for 4 h. Cell nuclei and CALR were detected by Hoechst and anti-CALR/FITC-conjugated anti-IgG antibodies staining, respectively. Flow cytometry analysis of $CALR^+$ tumor cells. HMGB1 release into culture supernatants of MNDV(GEM)-treated tumor cells was examined 24 h following treatment by ELISA. **(b)** Flow cytometry analysis of BMDC surface activation marker expression. **(c)** Quantification of TNF-α and IL-6 production from BMDC via ELISA. **(d)** *In vitro* test of MNDV(GEM)-induced human C33a cervical cancer apoptosis (Annexin-V and PI double-positive cells). **(e)** Quantification of TNF-α and IL-6 production from human macrophage-like THP-1 cells via ELISA. Data are presented as the means ± S.D. (n = 3). *P* values were analysed by one-way ANOVA and Tukey's tests. Source data are provided as a Source Data file.

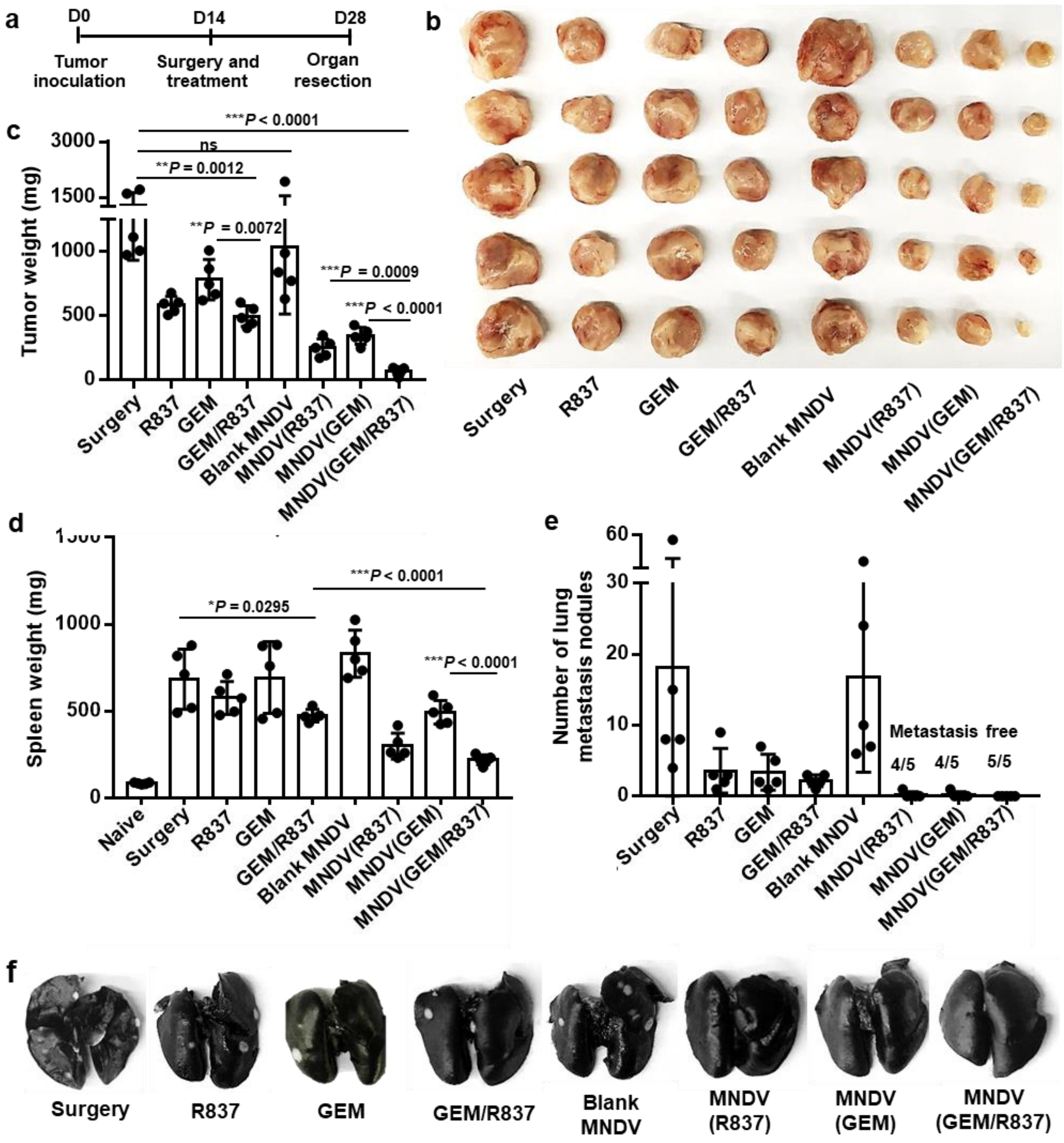

Supplementary Fig. 14. *In vivo* antitumor effect of MNDVs. **(a)** Schematic diagram for treatment schedule. **(b)** The recurrent tumor image, **(c)** tumor weight and **(d)** spleen weight at 14 days after surgery. **(e)** The mean numbers of macroscopically visible 4T1 breast cancer metastases in the lungs. **(f)** Representative images of lungs collected from mice. White nodules indicate metastatic tumors in the lungs. Data are presented as the means ± S.D. (n = 5). *P* values were analysed by Student's t-test. Source data are provided as a Source Data file.

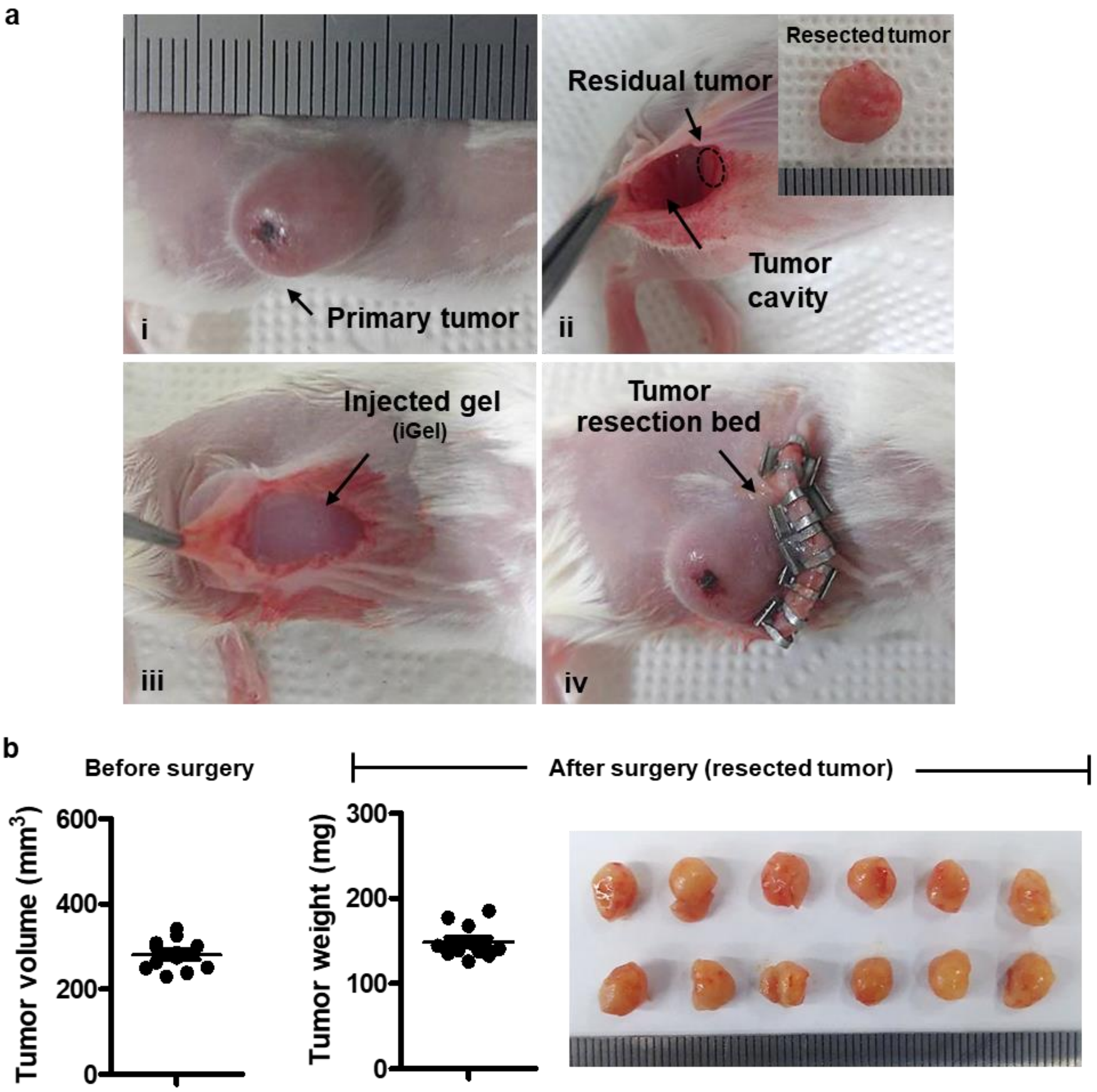

Supplementary Fig. 15. Tumor resection and the iGel injection procedure. **(a)** Surgical strategy: i) surgery was performed. ii) Tumor dissection mimicking incomplete tumor removal (approximately 90% of primary tumor was excised). iii) The gel was placed in the tumor cavity. iv) Wound closure. **(b)** Strategy to control tumor resection size. Tumors were measured and randomly allocated for each group. Tumors were removed (approximately 90% of the primary tumor). The removal tumors was confirmed by tumor weight and size.

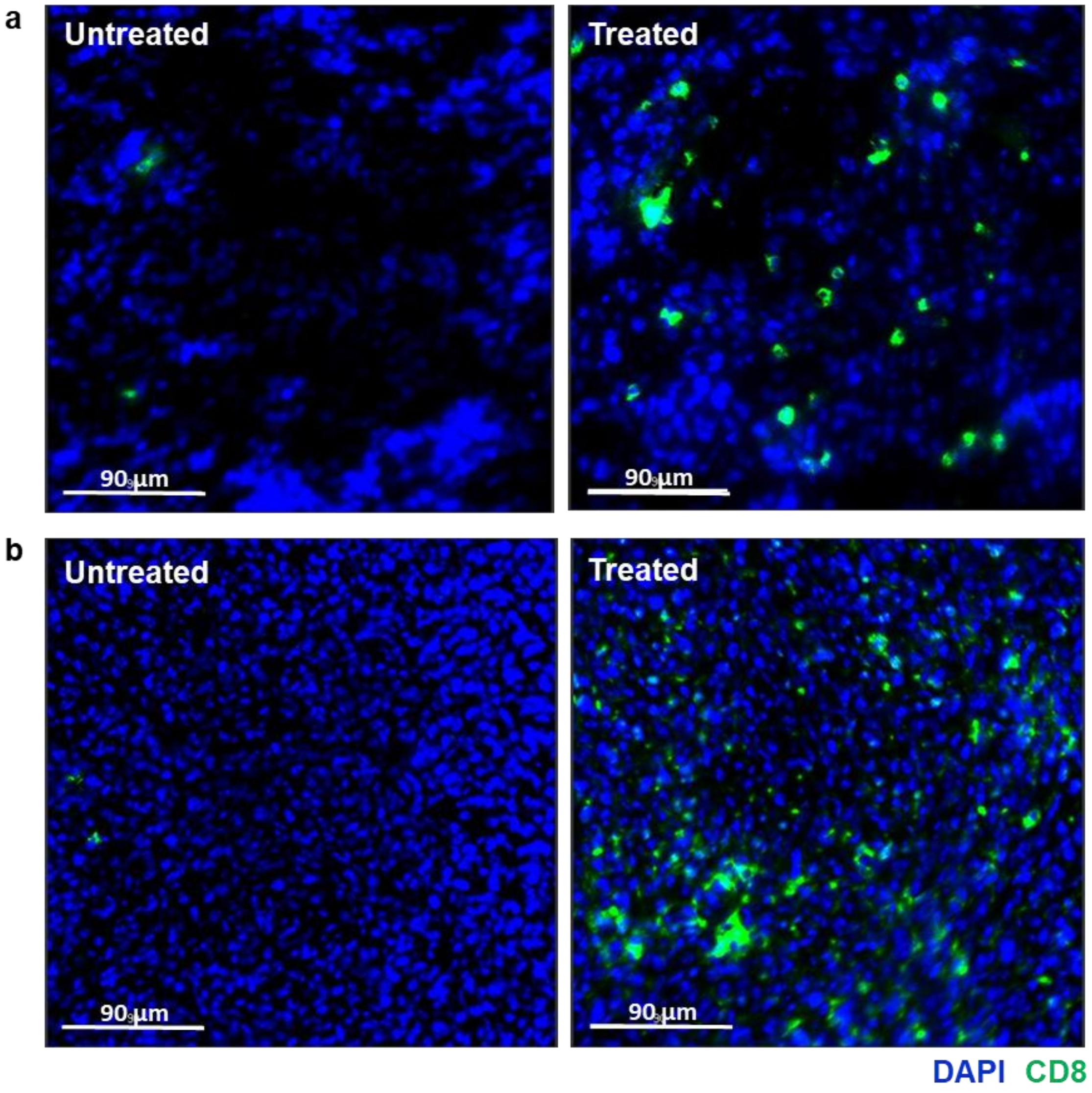

Supplementary Fig. 16. Representative immunofluorescence images of **(a)** 4T1 and **(b)** TC1 tumors showed an increase in $CD8^+$ cells in recurrent tumors 7 days after treatment with iGel. Scale bar is 90 µm (n = 5).

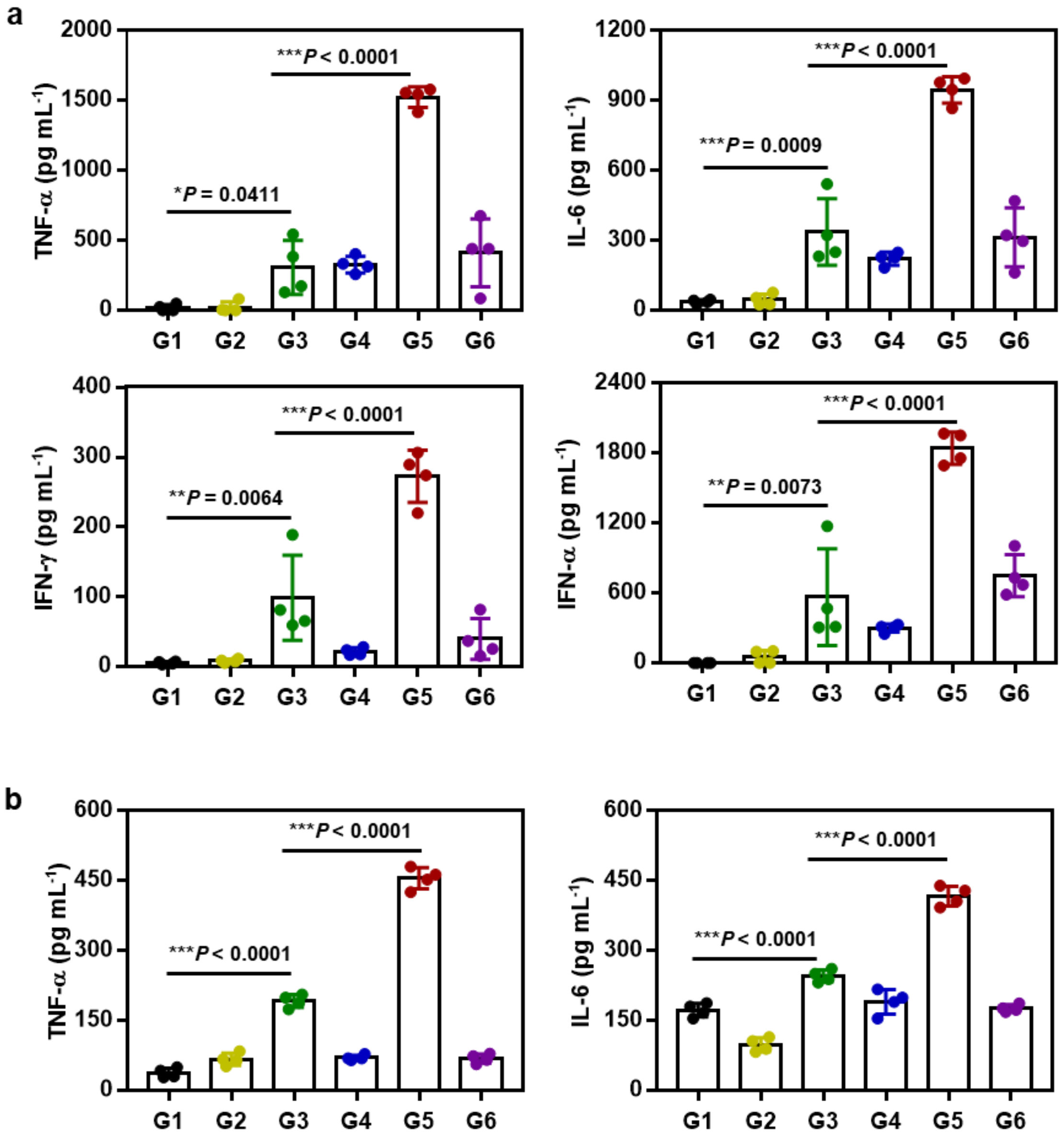

Supplementary Fig. 17. Pro-inflammatory cytokine in recurrent **(a)** 4T1 and **(b)** TC1 tumors at day 5 post-surgery and treatment with follows: G1, surgery only; G2, blank gel; G3, MNDV(GEM/R837)/CNL; G4, blank MNDV/clodronate-CNL; G5, MNDV (GEM/R837)/clodronate-CNL; and G6, MNDV(GEM/R837) /clodronate-ANL. Data are presented as the means ± S.D. (n = 4). The results are representative of one of two independent experiments. Statistical significance was calculated by one-way ANOVA and Tukey's tests. Source data are provided as a Source Data file.

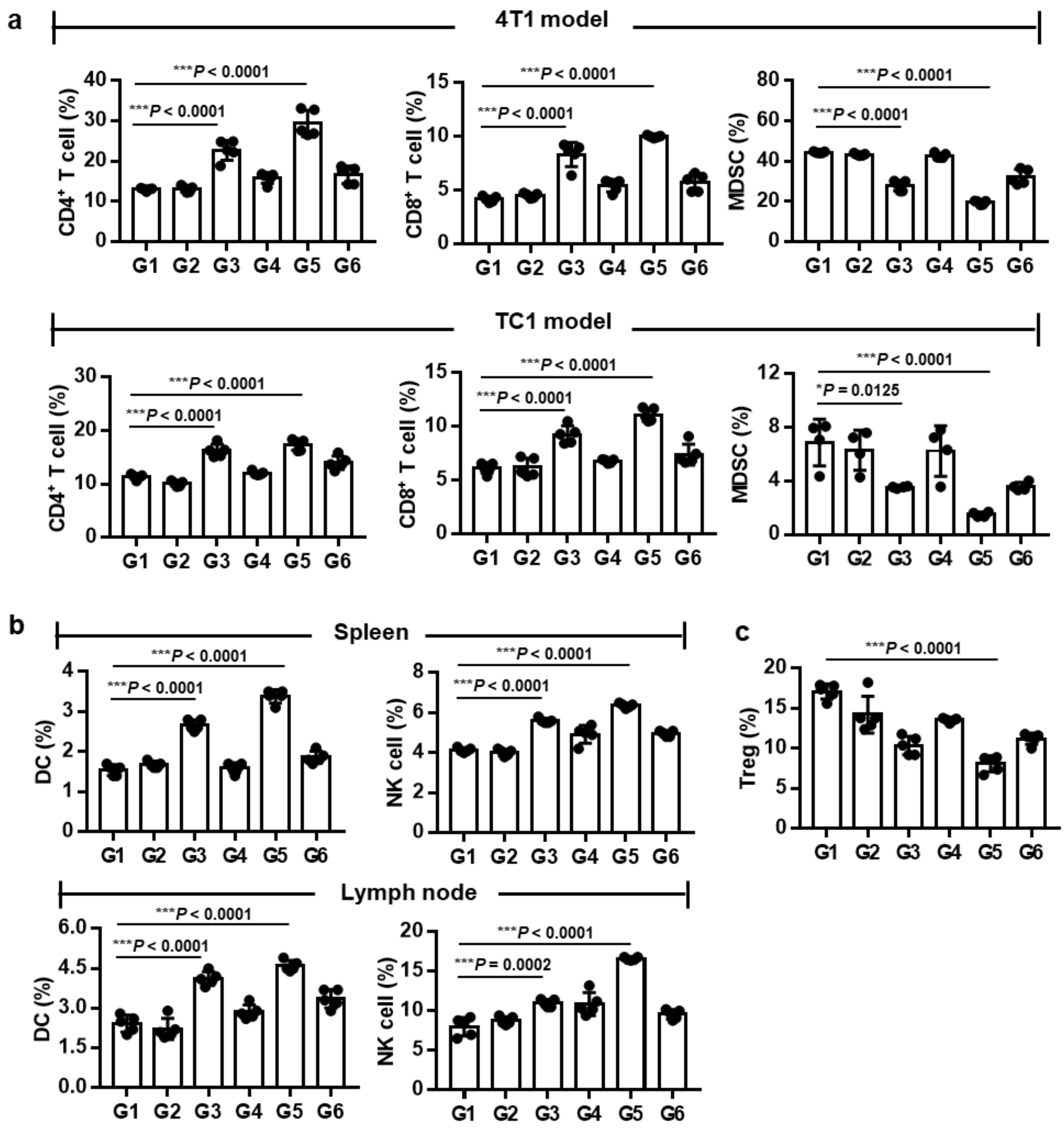

Supplementary Fig. 18. Infiltrating immune cell analysis in lymphoid organs at day 7 post-surgery and treatment with follows: G1, surgery only; G2, blank gel; G3, MNDV(GEM/R837)/CNL; G4, blank MNDV/clodronate-CNL; G5, MNDV (GEM/R837)/clodronate-CNL; and G6, MNDV(GEM/R837) /clodronate-ANL. **(a)** Fluorescence activated cell sorting analysis demonstrating infiltrating $CD4^+$, $CD8^+$ T cells, and MDSCs in the spleen at day 7 post-surgery in the 4T1 and TC1 tumor models. **(b)** Infiltrating innate immune cells (DC and NK cells) in the spleen and tumor draining lymph nodes in the 4T1 tumor model. **(c)** Infiltrating Treg cells ($CD25^+$ $FOXP3^+$ gated on $CD4^+$ cells) in tumor draining lymph nodes in the 4T1 tumor model. Data are presented as the means ± S.D. (n = 5). Statistical significance was calculated by one-way ANOVA and Tukey's tests. Source data are provided as a Source Data file.

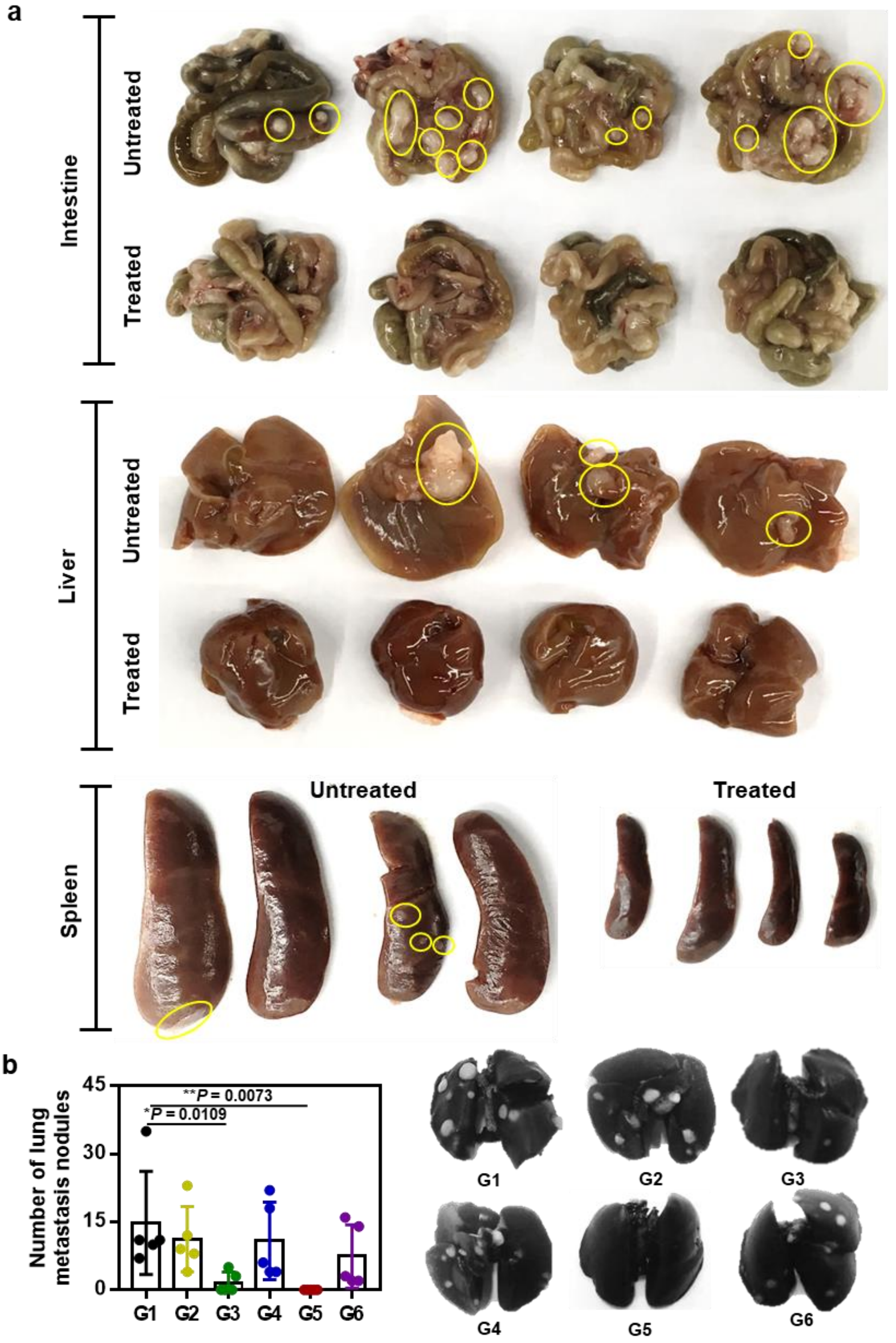

Supplementary Fig. 19. Inhibition of metastasis after iGel treatment. **(a)** Photographs of the intestine, liver and spleen collected from mice 21 days after tumor resection after treatment with iGel (G5 group) (n = 4). Yellow circles indicate metastatic tumors in the organ. **(b)** Inhibition of metastasis to the lung after treatment with follows: G1, surgery only; G2, blank gel; G3, MNDV(GEM/R837)/CNL; G4, blank MNDV/clodronate-CNL; G5, MNDV (GEM/R837)/clodronate-CNL; and G6, MNDV(GEM/R837) /clodronate-ANL. The mean numbers of macroscopically visible breast cancer metastases in the lungs collected from mice in the different treatment groups at day 10 after tumor resection (left). Representative images of lungs collected from mice (right). White nodules indicate metastatic tumors in the lungs. Data are presented as the means ± S.D. (n = 5). *P* values were analysed by Mann-Whitney test. Source data are provided as a Source Data file.

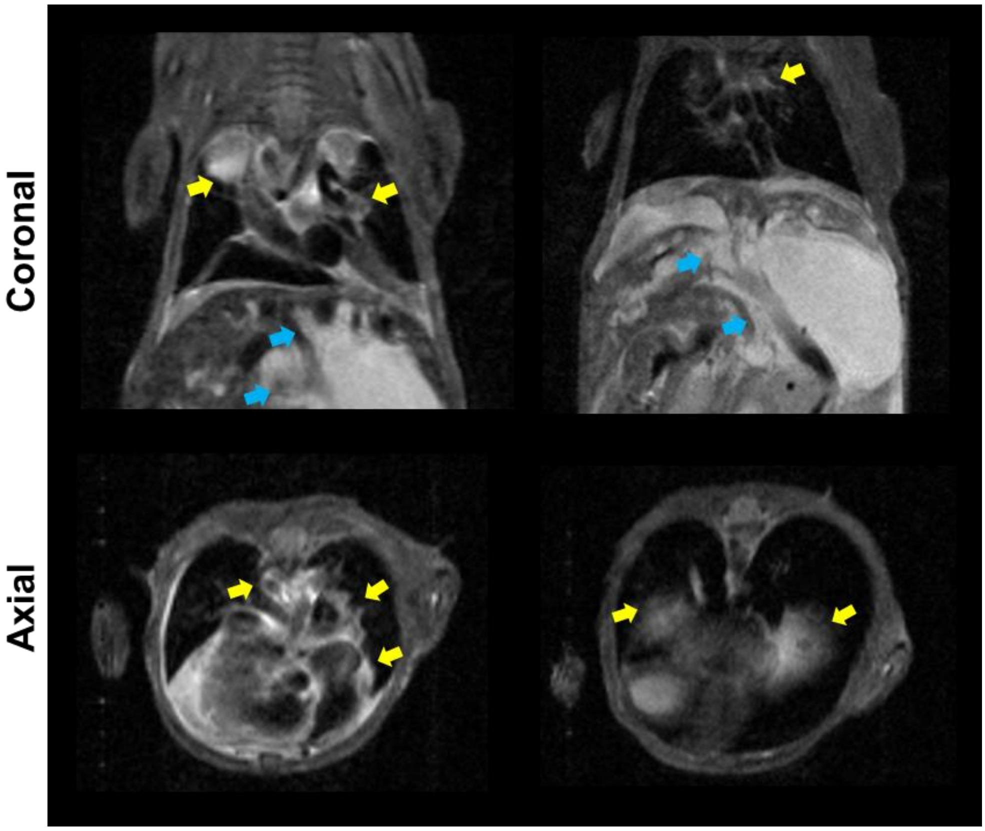

Supplementary Fig. 20. MRI scan images showing tumor metastasis in untreated mice. Yellow arrows in coronal and axial images indicate the tumors in lung. Blue arrows in coronal image of untreated group indicate tumors in other body parts. Another hyper-intense white signal that is not indicated by arrows is related to massive ascites, which reflected the aggressive features of peritoneal spread tumor (n = 3).

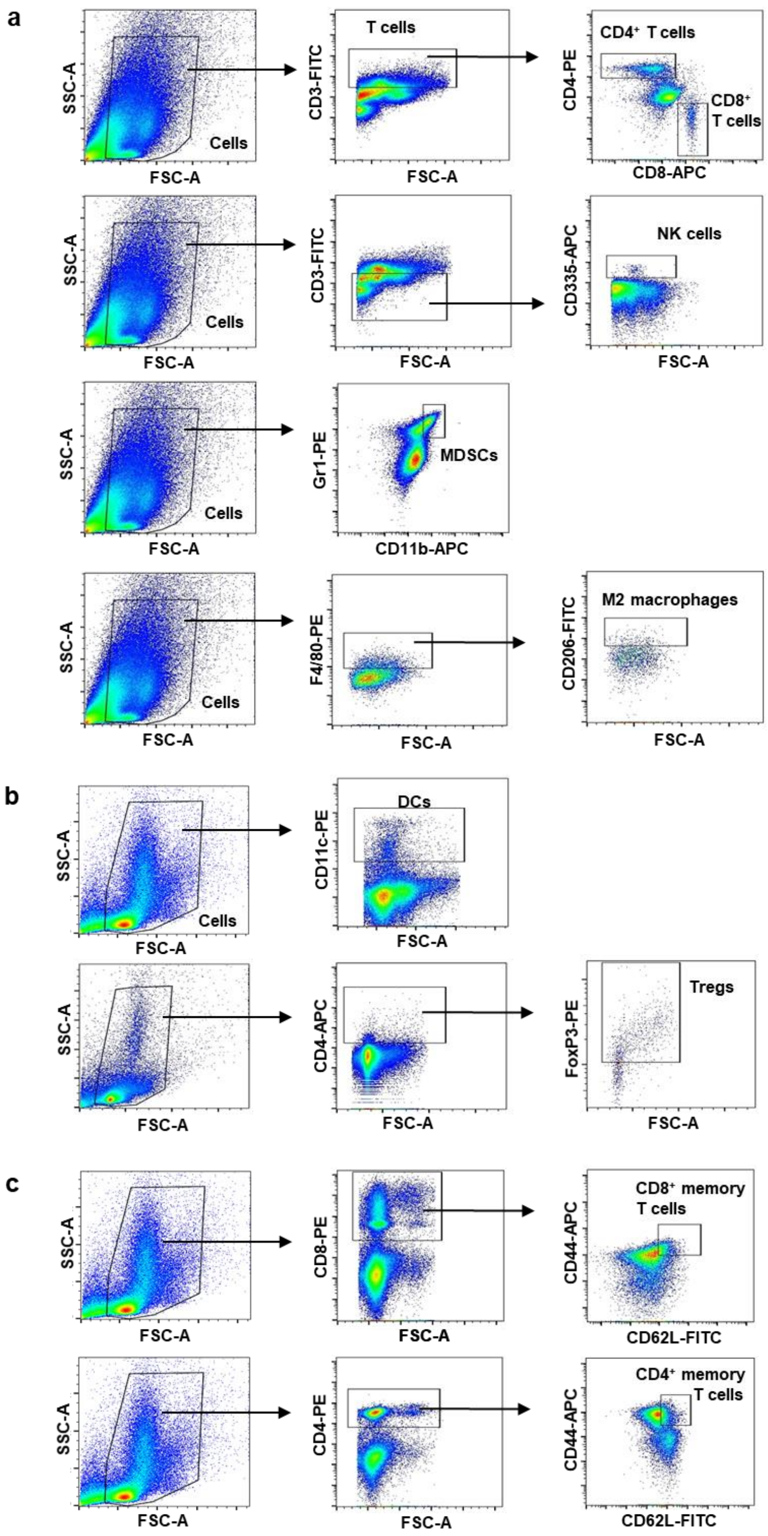

Supplementary Fig. 21. Gating scheme for flow cytometric analysis of immune cell populations. **(a)** $CD4^+$ and $CD8^+$ T cells, NK cells, MDSCs and M2 macrophages presented on Fig. 4c, 5c, 8e and 8j. **(b)** DC and Tregs presented on Supplementary Fig. 18. **(c)** $CD8^+$ and $CD4^+$ memory T cells presented on Fig. 7a. Initial cell populations were gated for a live population using FSC and SSC plot of cell only sample. The gating strategy for all samples was set to remove cell debris, dead cells (small FSC and SSC), and large clumps or aggregates of cells (large FSC and SSC). The percentage reflects the ratio of specific immune cell to the total live population.

Supplementary Table

Neutral Lipid	Cationic Lipid	Clodronate	Molar ratio	Size (nm)	Zeta (mV)	Encapsulation efficiency (%)
DOPE	DOTAP	500 µg	1 : 1	105.4 ± 9.2	36.9 ± 1.9	90.3 % (451.4 µg)
CH	DOTMA	500 µg	1 : 1	83.3 ± 13.4	43.1 ± 1.1	94.0 % (469.8 µg)
DOPE	DOTMA	500 µg	1 : 1	77.9 ± 7.5	57.3 ± 3.9	97.7 % (488.3 µg)
DOPE	DOTMA	1000 µg	1 : 1	107.5 ± 6.8	36.3 ± 1.3	82.4 % (823.7 µg)
DOPE	DOTMA	2000 µg	1 : 1	← *aggregation* →		

Supplementary Table 1 Optimization of clodronate loaded cationic nanoliposome formulation
