# Supplementary material for: Syringeable immunotherapeutic nanogel reshapes tumor microenvironment and prevents tumor metastasis and recurrence
Source: Nat Commun. 2019 Aug 20;10:3745. doi: 10.1038/s41467-019-11730-8 (PMC6702226; doi:10.1038/s41467-019-11730-8)
Supplement: Supplementary file 2 — Reporting Summary [file 41467_2019_11730_MOESM2_ESM.pdf]

nature research

Corresponding author(s): Yong Taik Lim, Tae Heung Kang, Yeong-Min Park

Last updated by author(s): Jul 25, 2019

Reporting Summary

Nature Research wishes to improve the reproducibility of the work that we publish. This form provides structure for consistency and transparency in reporting. For further information on Nature Research policies, see Authors & Referees and the Editorial Policy Checklist.

Statistics

For all statistical analyses, confirm that the following items are present in the figure legend, table legend, main text, or Methods section.

n/a	Confirmed	
☐	☒	The exact sample size (*n*) for each experimental group/condition, given as a discrete number and unit of measurement
☐	☒	A statement on whether measurements were taken from distinct samples or whether the same sample was measured repeatedly
☐	☒	The statistical test(s) used AND whether they are one- or two-sided<br>*Only common tests should be described solely by name; describe more complex techniques in the Methods section.*
☐	☒	A description of all covariates tested
☐	☒	A description of any assumptions or corrections, such as tests of normality and adjustment for multiple comparisons
☐	☒	A full description of the statistical parameters including central tendency (e.g. means) or other basic estimates (e.g. regression coefficient) AND variation (e.g. standard deviation) or associated estimates of uncertainty (e.g. confidence intervals)
☐	☒	For null hypothesis testing, the test statistic (e.g. *F*, *t*, *r*) with confidence intervals, effect sizes, degrees of freedom and *P* value noted<br>*Give P values as exact values whenever suitable.*
☒	☐	For Bayesian analysis, information on the choice of priors and Markov chain Monte Carlo settings
☒	☐	For hierarchical and complex designs, identification of the appropriate level for tests and full reporting of outcomes
☒	☐	Estimates of effect sizes (e.g. Cohen's *d*, Pearson's *r*), indicating how they were calculated

Our web collection on statistics for biologists contains articles on many of the points above.

Software and code

Policy information about availability of computer code

Data collection	Flow cytometry data was collected using BD Accuri C6 Plus and BD FACSDiva software (v8.0.1.1). SoftMax Pro 5.0 was using for plate based absorbance data. Simple PCI 6 was used for in vivo fluorescence imaging and DeltaVision softWoRx was used for in vitro fluorescence imaging. UVProbe 2.62 was used for in vitro release data. Gatan DigitalMicrograph was used for TEM. ParaVision v5.1 was used for MRI. ELSZ-1000 was used for DLS/zeta. TRIOS software was used for rheological data.
Data analysis	Microsoft Excel and Graphpad Prism 7 were used for statistical analysis and plotting. FlowJo VX was used for flow cytometry analysis. Simple PCI 6 and ImageJ software were used to analyze fluorescent image and MNDV diameter. For MRI image analysis ParaVision v5.1 was used.

For manuscripts utilizing custom algorithms or software that are central to the research but not yet described in published literature, software must be made available to editors/reviewers. We strongly encourage code deposition in a community repository (e.g. GitHub). See the Nature Research guidelines for submitting code & software for further information.

Data

Policy information about availability of data

All manuscripts must include a data availability statement. This statement should provide the following information, where applicable:

- Accession codes, unique identifiers, or web links for publicly available datasets
- A list of figures that have associated raw data
- A description of any restrictions on data availability

The data that support the findings of this study are available from the corresponding author upon reasonable request.

Field-specific reporting

Please select the one below that is the best fit for your research. If you are not sure, read the appropriate sections before making your selection.

☒ Life sciences ☐ Behavioural & social sciences ☐ Ecological, evolutionary & environmental sciences

For a reference copy of the document with all sections, see nature.com/documents/nr-reporting-summary-flat.pdf

Life sciences study design

All studies must disclose on these points even when the disclosure is negative.

Sample size	Details regarding the sample size of all experiments are provided in figure legends. Sample size were estimated to achieve about 90% power for detection of significant differences in tumor volume between groups based on means and standard deviations in preliminary studies. They were consistent with sample size of previously reported results in other studies.
Data exclusions	No data were excluded.
Replication	The replicated experiments were noted in the manuscript as one of x independent experiments. Experiments were repeated and experimental findings were reproducible. The conclusions derived from the data in each experiment were consistent.
Randomization	For in vitro test, samples were randomly allocated to corresponding experimental groups. For in vivo test, mice were inoculated tumor at the same time and then randomly assigned to a group for similar average tumor sizes. For tumor resection procedure, the remaining tumor was precisely controlled as shown detail in method and supplementaly information. Mice were allocated randomly to each treatment group.
Blinding	Survival test, tumor volume measurement and imaging experiments to test the efficacy of each treatment groups were conducted by independent researchers who were unaware of the treatment conditions.

Reporting for specific materials, systems and methods

We require information from authors about some types of materials, experimental systems and methods used in many studies. Here, indicate whether each material, system or method listed is relevant to your study. If you are not sure if a list item applies to your research, read the appropriate section before selecting a response.

Materials & experimental systems

n/a	Involved in the study	
☐	☒	Antibodies
☐	☒	Eukaryotic cell lines
☒	☐	Palaeontology
☐	☒	Animals and other organisms
☒	☐	Human research participants
☒	☐	Clinical data

Methods

n/a	Involved in the study	
☒	☐	ChIP-seq
☐	☒	Flow cytometry
☒	☐	MRI-based neuroimaging

Antibodies

Antibodies used

The following antibodies were used for flow cytometry:
Anti-CD3: FITC (clone: 17A2, catalogue: 100204, Biolegend)
Anti-CD4: PE (clone: RM4-4, catalogue: 116006, Biolegend)
Anti-CD4: APC (clone:RM4-4, catalogue: 116014, Biolegend)
Anti-CD8: APC (clone: 53-6.7, catalogue: 100712, Biolegend)
Anti-CD8: PE (clone: 53-6.7, catalogue: 100708, Biolegend)
Anti-CD40: FITC (clone: 3/23, catalogue: 124607, Biolegend)
Anti-CD80: FITC (clone:16-10A1, catalogue: 104705, Biolegend)
Anti-CD11b: APC (clone: M1/70, catalogue: 101212, Biolegend)
Anti-CD11c: APC (clone:N418, catalogue: 117309, Biolegend)
Anti-CD11c: PE (clone:N418, catalogue: 117307,Biolegend)
Anti-Gr1: PE (clone: RB6-8C5, catalogue: 108408)
Anti-FOXP3: PE (clone:MF-14, catalogue: 126404, Biolegend)
Anti-CD206: FITC (clone: C068C2, catalogue: 141704, Biolegend)
Anti-F4/80: PE (clone: BM8, catalogue: 123110, Biolegend)
Anti-CD335: APC (clone: 29A1.4, catalogue: 137602, Biolegend)
Anti-CD44: FITC (clone: IM7, catalogue: 103005, Biolegend)
Anti-CD62L: APC (clone: MEL-14, catalogue: 104412, Biolegend)
Anti-CD45: FITC (clone:30-F11, catalogue: 103107,Biolegend)
Anti-PD-1: PE (clone: 29F.1A12, catalogue: 135205, Biolegend)

Anti-PD-L1: APC (clone: 10F.9G2, catalogue: 124311, Biolegend)

The following antibodies were used for depletion of immune cells:
InvivoMAb anti-mouse CD8 (clone: 2.43, catalogue: BE0061, Bioxcell)
InvivoMAb anti-mouse CD4 (clone: GK1.5, catalogue: BE0003-1, Bioxcell)
Purified anti-Asialo-GM1 antibody (clone: Poly21460, catalogue: 146002, Biolegend)

The following antibodies were used for combination therapy:
InvivoMAb anti-mouse PD-1 (clone: RMP1-14, catalogue: BE0146, Bioxcell)
InvivoMAb anti-mouse PD-L1 (clone: 10F.9G2, catalogue: BE0101, Bioxcell)

The following antibodies were used for in situ immunofluorescence of recurring tumor:
Anti-CD8 antibody (clone: EPR21769, catalogue: ab217344, Abcam)
Goat Anti-Rabbit IgG H&L: Alexa fluor 488 (catalogue: ab150077, Abcam)

The following antibodies were used for detection of immunogenic cell death:
Anti-calreticulin antibody (catalogue: ab2907, Abcam)
Goat Anti-Rabbit IgG (H+L): FITC (catalogue: 111-095-003, Jackson ImmunoResearch)

All the antibodies used in the experiments were purchased from commercial sources, and the dilution and use of the antibodies were consulted to the manufacturer's recommendations.

Validation

The antibodies for flow cytometry were validated by Biolegend using bone marrow cells or splenocytes from C57BL/6 mice, with related data shown on the manufacturer website. The validation of therapeutic antibodies were performed by SDS-PAGE by Bioxcell, with relevant data presented on the manufacturer website. Additional validation of both therapeutic and flow cytometric antibodies was not performed by the authors.

Eukaryotic cell lines

Policy information about cell lines

Cell line source(s)

4T1 breast cancer cells and TC1 cervical cancer cells were used and obtained from American Type Culture Collection (ATCC, Manassas, VA, USA). The human monocyte cell line THP-1 and human cervical cancer cell line C33a were obtained from ATCC.

Authentication

The cell lines were morphologically confirmed according to the information provided by ATCC

Mycoplasma contamination

All cell lines were tested for mycoplasma contamination. Mycoplasma contamination was not found

Commonly misidentified lines
(See ICLAC register)

No commonly misidentified cell lines were used.

Animals and other organisms

Policy information about studies involving animals; ARRIVE guidelines recommended for reporting animal research

Laboratory animals

All of the animal handling and studies were conducted based on the guidelines and approval by the Institutional Animal Care and Use Committee (IACUC) of Sungkyunkwan University School of Medicine, which is accredited by the Association for Assessment and Accreditation of Laboratory Animal Care International (AAALAC International) and abides by the Institute of Laboratory Animal Resources (ILAR) guide. 6weeks BALB/c and C57BL/6 female mice were purchased from Orient Bio, Seongnam, South Korea.

Wild animals

This study did not involve wild animals.

Field-collected samples

This study did not involve field-collected samples.

Ethics oversight

All of the animal handling and studies were conducted based on the guidelines and approval by the Institutional Animal Care and Use Committee (IACUC) of Sungkyunkwan University School of Medicine, which is accredited by the Association for Assessment and Accreditation of Laboratory Animal Care International (AAALAC International) and abides by the Institute of Laboratory Animal Resources (ILAR) guide.

Note that full information on the approval of the study protocol must also be provided in the manuscript.

Flow Cytometry

Plots

Confirm that:

☒ The axis labels state the marker and fluorochrome used (e.g. CD4-FITC).

☒ The axis scales are clearly visible. Include numbers along axes only for bottom left plot of group (a 'group' is an analysis of identical markers).

☒ All plots are contour plots with outliers or pseudocolor plots.

☒ A numerical value for number of cells or percentage (with statistics) is provided.

Methodology

Sample preparation	Tumors, spleen and tumor-draining lymph nodes were harvested from sacrificed mice. The tumors and lymph nodes were cut into small pieces and resuspended in collagenase D in DMEM (1 mg/mL). The solutions were incubated for 1 h at 37°C on a shaker (90 rpm) and then filtered through a 70-µm Falcon cell strainer. The supernatant from the digested tumor tissues was collected, centrifuged at 490 × g for 5 min, and resuspended. The spleen was mechanically dissociated and resuspended in DMEM. The suspension was filtered through a 70-µm Falcon cell strainer, centrifuged and resuspended. Erythrocytes were lysed with red blood cell lysis buffer for 5 min at 37°C. Cell suspensions were prepared as described above and then stained with the antibodies. The cells were then washed twice and analysed using flow cytometer.
Instrument	BD Acurri C6 cytometer, BD FACSCelesta and BD FACSCanto II
Software	BD C6 Plus software, FACS DIVA and FlowJo VX
Cell population abundance	Flow cytometry was used for quantification only. No post-sort fractions were collected.
Gating strategy	Initial cell populations were gated for a live population using FSC and SSC plot of cell only sample. The gate was set to remove cell debris and dead cells (small FSC and SSC) and large clumps or aggregates of cells (large FSC and SSC) and used across all samples. This live population was then further gated as shown in Supplementary Fig. S21.

☒ Tick this box to confirm that a figure exemplifying the gating strategy is provided in the Supplementary Information.
